# Identification and validation of a novel CD8+ T cell-associated prognostic model based on ferroptosis in acute myeloid leukemia

**DOI:** 10.3389/fimmu.2023.1149513

**Published:** 2023-04-17

**Authors:** Ge Jiang, Peng Jin, Xiao Xiao, Jie Shen, Ran Li, Yunxiang Zhang, Xiaoyang Li, Kai Xue, Junmin Li

**Affiliations:** ^1^ State Key Laboratory of Medical Genomics, Shanghai Institute of Hematology, National Research Center for Translational Medicine at Shanghai, Ruijin Hospital Affiliated to Shanghai Jiao Tong University School of Medicine, Shanghai, China; ^2^ Department of Orthopedic, Shanghai Tenth People’s Hospital, Tongji University School of Medicine, Shanghai, China

**Keywords:** CD8+ T cell, acute myeloid leukemia (AML), prognosis, ferroptosis, European Leukemia Net (ELN) 2017

## Abstract

Acute myeloid leukemia (AML) is a highly aggressive cancer with great heterogeneity and variability in prognosis. Though European Leukemia Net (ELN) 2017 risk classification has been widely used, nearly half of patients were stratified to “intermediate” risk and requires more accurate classification *via* excavating biological features. As new evidence showed that CD8+ T cell can kill cancer cells through ferroptosis pathway. We firstly use CIBERSORT algorithm to divide AMLs into CD8+ ^high^ and CD8+ ^low^ T cell groups, then 2789 differentially expressed genes (DEGs) between groups were identified, of which 46 ferroptosis-related genes associated with CD8+ T cell were sorted out. GO, KEGG analysis and PPI network were conducted based on these 46 DEGs. By jointly using LASSO algorithm and Cox univariate regression, we generated a 6-gene prognostic signature comprising *VEGFA*, *KLHL24*, *ATG3*, *EIF2AK4*, *IDH1* and *HSPB1*. Low-risk group shows a longer overall survival. We then validated the prognostic value of this 6-gene signature using two independent external datasets and patient sample collection dataset. We also proved that incorporation of the 6-gene signature obviously enhanced the accuracy of ELN risk classification. Finally, gene mutation analysis, drug sensitive prediction, GSEA and GSVA analysis were conducted between high-risk and low-risk AML patients. Collectively, our findings suggested that the prognostic signature based on CD8+ T cell-related ferroptosis genes can optimize the risk stratification and prognostic prediction of AML patients.

## Introduction

Acute myeloid leukemia (AML), which is highly heterogeneous in adults, represents the most common hematologic malignancy worldwide (1–3). The American Cancer Society has reported a diagnoses rate of more than 20000 new cases of AML in 2021 in the United States alone, while the 5-year overall survival rate is lower than 30% (4). A recent, advanced risk stratification model for AML based on risk classification by the European Leukemia Net (ELN) 2017 categorizes AML patients into three groups for prediction of treatment responses and prognosis (5, 6). Despite its extensive use, the increasing availability of multi-omics data presents an opportunity for improvements to the ELN2017 model through the incorporation of molecular expression data (7–9), which could facilitate higher accuracy patient stratification and therapeutic decision-making. In particular, the use of transcriptomic data that contain the underlying molecular basis responsible for AML pathophysiology could further improve prognosis and enable the identification of novel therapeutic targets.

The strategies for treating AML have remained relatively unchanged over the past three decades. While the “7+3” combined chemotherapy regimen reportedly leads to complete remission in 60-80% in patients younger than 60-years-old and 40%-60% in patients older than 60 years of age (10), in addition to the durable complete remission in the limited group of patients eligible for allogeneic hematopoietic stem cell transplantation (HSCT), both HSCT and chemotherapy treatments have long been associated with high relapse rates (11). Emerging tumor immunotherapy approaches that rely on T cell activation (including CAR-T, TCR-T, or cancer vaccines, etc.) have also shown promise for improved treatment response and clinical outcomes in AML patients (12). These immunotherapeutic approaches rely on the potent effects of CD8+ T cell activation to combat hematopoietic malignancies, since these cells perform essential functions in mediating tumor adaptive immunity (13, 14). Generally, CD8+ T cells exert their killing effects through two main pathways for inducing apoptosis in tumor cells: granzyme-perforin and Fas-FasL (15).

In addition to the canonical apoptotic routes, ferroptosis is an iron-dependent program for regulated cell death (RCD) induced by oxidative disruption of the intracellular microenvironment, which has been implicated as a determining factor in pathogenic progression and treatment response in AML (16, 17). Ferroptosis-related genes can therefore enhance predictive accuracy in AML prognosis over that of apoptosis-related genes since cancer cells exhibit marked sensitivity to ferroptosis due to the necessity of related factors such as iron accumulation and fatty acid synthesis, among others, for tumor growth (16, 18, 19).

Interestingly, correlation between CD8+ T cell and ferroptosis has been unveiled recently. For example, Wang et al. provided direct evidence that CD8+ T cells can mediate resistance to cancer cells *via* the ferroptosis pathway (20). Another research conducted by Liao et al. further proved that polyunsaturated fatty acids (PUFAs) and CD8+ T cell-derived interferon (IFN) γ work together as a natural ferroptosis inducer (FIN) to cause tumor ferroptosis and boost anti-tumor immunity (21).

Inspired by the correlation, our work innovatively investigated CD8+ T cell-related ferroptosis prognostic model in AML, which has a potential to optimize ELN 2017 classification and enable the identification of new and effective therapeutic strategies.

## Materials and methods

### Patient sample collection and RNA-sequencing

A total of 157 bone marrow (BM) aspirates were collected from 157 *de novo* AML patients diagnosed between June 2019 and September 2020 at Ruijin Hospital affiliated to Shanghai Jiao Tong University School of Medicine. Following the Declaration of Helsinki, the Institutional Review Board of Ruijin Hospital approved the collection of the specimens, and all patients provided written informed consent for specimen collection and research. Total RNA was extracted and RNA-seq libraries were constructed using the TruSeq RNA Sample Preparation Kit v2 (Illumina, San Diego, CA, USA). Paired-end sequencing (150-bp) was performed on the NovaSeq 6000 platform (Illumina). Adapter sequences were trimmed from raw sequencing reads using Trim-galore and then aligned with STAR aligner. Cleaned reads were quantified with HT-Seq count through mapping to the GRCh38 human reference genome assembly. Gene expression estimates were normalized to Transcripts Per Kilobase of exon model per Million mapped reads (TPM) using a customed script. The cohort was named ‘RJAML’ below.

### Data collection

Gene expression and mutation data from the TCGA-LAML cohort (N=151) were obtained from the GDC data portal. Similarly, the gene expression profiles of the GSE12417 (GPL570, N=78) and GSE71014 (N=104) datasets were downloaded from the GEO database. Subsequently, we obtained 259 ferroptosis-related genes from FerrDb DATABASE (http://www.zhounan.org/ferrdb/index.html).

### Estimation of immune cell infiltration

CIBERSORT algorithm (22) was used to examine the relative proportions of the 22 immune infiltrating cell types including CD8+ T cell. Correlation between gene expression and immune cell population data were analyzed using the Spearman correlation method, with p < 0.05 regarded statistically significant.

### Pathway enrichment analysis of CD8+ T cell-related ferroptosis genes

To explore the potential biological functions and pathways related to CD8+ T cell-related ferroptosis genes, DEG analysis was performed using the edgeRR package between CD8+ ^high^ and CD8+ ^low^ T cell groups. A cutoff of |logFC| > 1, and *p*< 0.05 was used to define DEGs, of which 46 DEGs were related to ferroptosis. Gene Ontology (GO) and Kyoto Encyclopedia of Genes and Genomes (KEGG) were used to make a comprehensive investigation for the CD8+ T cell-related ferroptosis genes based on “Cluster Profiler” (R3.6). GO and KEGG enrichment pathways with both *p*- and *q*-value< 0.05 were considered as significant.

### Construction and validation of a prognostic ferroptosis-related gene signature

Univariate cox analysis of overall survival (OS) was performed to screen for ferroptosis-related genes with potential prognostic value. To construct a prognostic model that minimized the risk of overfitting, we used LASSO-penalized Cox regression analysis based on partial likelihood deviance and lambda values, with the value of lambda corresponding to the lowest partial likelihood deviance. After normalizing the expression values of each specific gene, a risk score formula was generated for each patient as follows,


$$\begin{array}{l} Risk\;Score\;=VEGFA*\;\left(-0.136\right)\;+KLHL24*\;\left(-0.034\right)\\ \;+ATG3*\;0.031\;+EIF2AK4*\;0.146\\ \;+IDH1*\;0.186+HSPB1*\;0.297 \end{array}$$

and scores were weighted with the regression coefficient estimated by the lasso regression analysis. Based on the above risk score formula, patients were assigned to high-risk or low-risk groups, and the median risk score was used as the cut-off value. The difference in overall survival (OS) between the two groups was assessed by Kaplan-Meier and compared using log-rank statistics. The role of risk scores in predicting patient prognosis was examined using lasso regression analysis and stratified analysis. The accuracy of model predictions was examined using ROC curves.

### Improving the European Leukemia Net 2017 risk stratification system

According to the ELN2017 classification system and the 6-gene signature, we refined ELN2017 risk classification by reclassifying patients with favorable ELN and low 6-gene scores as a favorable group; those with favorable ELN and high 6-gene scores or intermediate ELN and low 6-gene scores were included in the intermediate group, while the other three subgroups were included in the adverse group.

### Drug sensitivity analysis

We used the R package ‘pRRophetic’ to predict the chemosensitivity of each tumor sample *via* the largest pharmacogenomic database (Genomics Database for Cancer Drug Sensitivity, GDSC). The IC50 estimations for each individual chemotherapeutic drug treatment were calculated using the regression method, and ten cross-validations with the GDSC training set were carried out to test the accuracy of the regression and prediction. All parameters, including a ‘combat’ to eliminate batch effects and an average of duplicate gene expression, were set to their default values.

### Gene set enrichment analysis

Gene sets were filtered using a minimum and maximum gene set size of 20 and 500 genes, respectively. After performing 1,000 alignments, enriched gene sets were obtained based on a *p*< 0.05 and a false discovery rate (FDR) value of 0.25. At last, significantly enriched GO terms and KEGG pathways were demonstrated.

### Statistical analysis

Survival curves were generated by the Kaplan-Meier method and significance was determined by log-rank tests. Multivariate analysis was carried out using the Cox proportional-hazards model. All statistical analyses were performed using the R software package (version 3.6). All statistical tests were two-sided with *p*< 0.05 considered statistically significant.

## Results

### Identification of CD8+ T cell-related ferroptosis genes in AML

**Figure 1:**
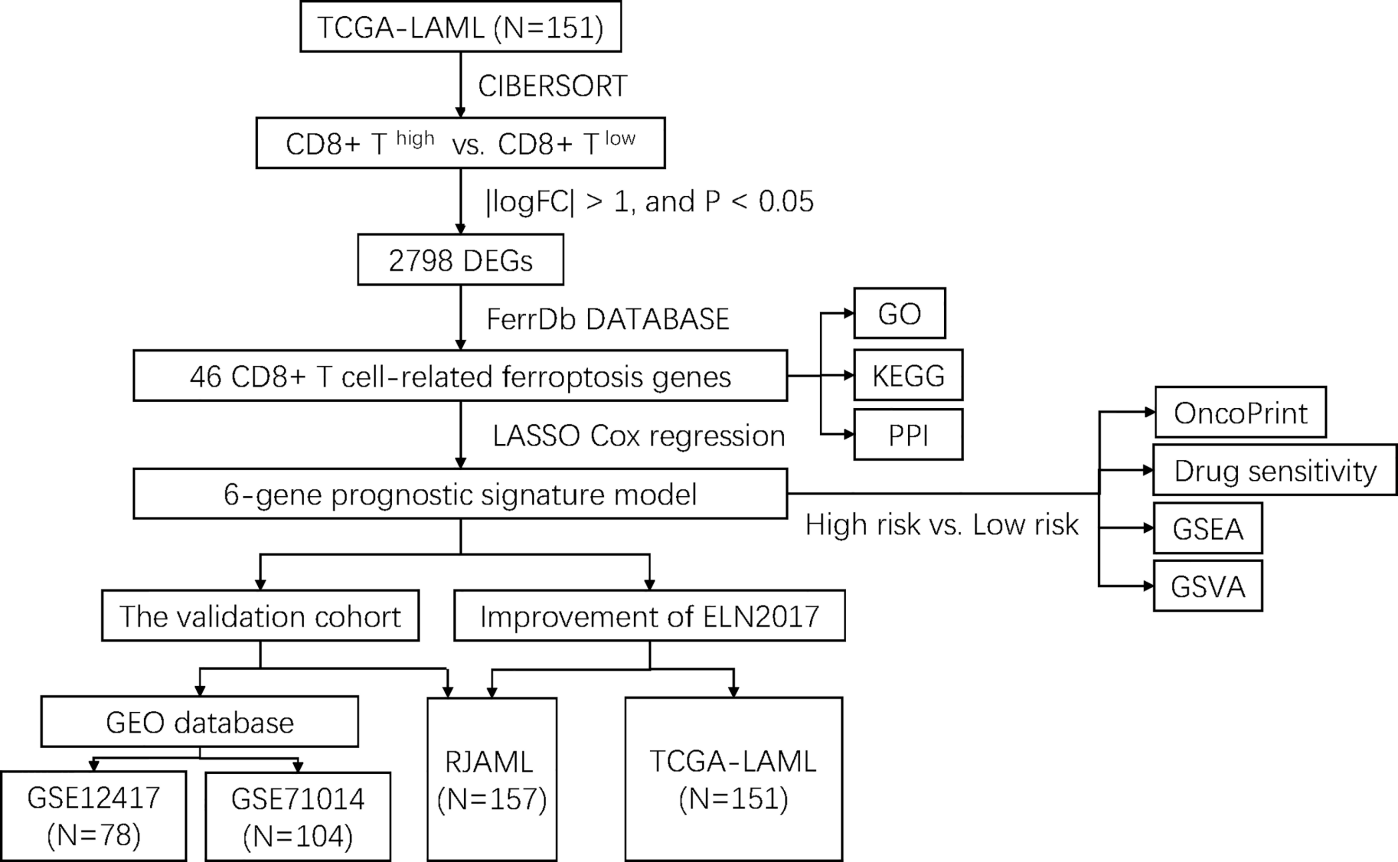
Flowchart of study design.

**Figure 2:**
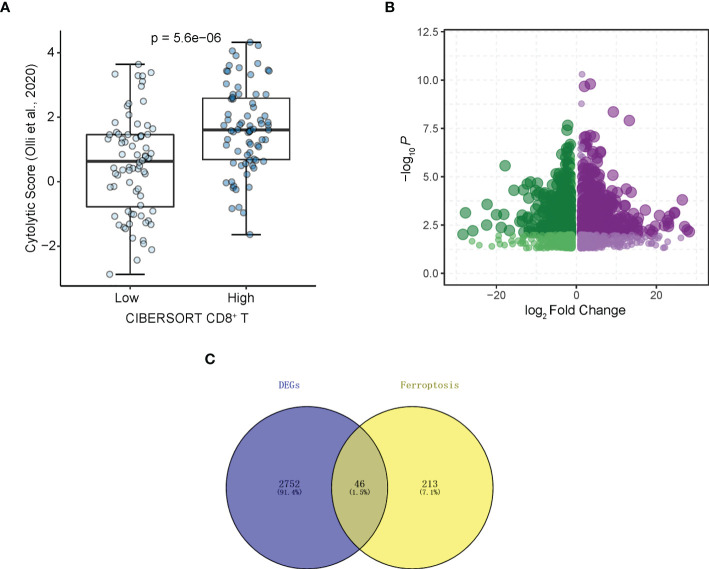
Identifying CD8+ T cell-related ferroptosis genes in AML. **(A)** Cytolytic scores (Olli et al., 2020) of patients from CD8+ T cell high and low groups divided by CIBERSORT. **(B)** Volcano plot of DEGs. Plots represents differentially expressed genes between CD8+ T cell high and low groups. **(C)** DEGs associated with ferroptosis.

The flowchart of the entire study is shown in **Figure 1**. Firstly, we quantified the proportion of immune infiltrating cells for each patient using the CIBERSORT algorithm and divided the samples (TCGA-LAML cohort, N=151) into CD8+ ^high^ and CD8+ ^low^ T cell groups based on the median percentage of infiltrating CD8+ T cells. Patients with high infiltration of CD8+ T cells had significantly higher cytolytic scores, which reflected cytolytic cell abundance, as previous reported (**Figure 2A**) (23). **Figure 1S** demonstrates the accordance of flow cytometry enumeration with CIBERSORT deconvolution for 6 AML samples (The experimental method is described in the **Supplementary Data Sheet 1**). We then performed differential expression analysis between high and low CD8+ T cell infiltration groups, which revealed a total of 2,798 differentially expressed genes (DEGs, |logFC| > 1, and *p*< 0.05), including 1,415 up-regulated and 1,383 down-regulated genes (**Figure 2B**). Notably, 46 of the DEGs were associated with ferroptosis (**Figure 2C**). Supplementally, the expression of these 46 CD8+ T cell-related ferroptosis genes in the CD8+ ^high^ and CD8+ ^low^ T cell groups is presented in the **Supplementary Data Sheet 2**. 

### Molecular functions and pathways enriched by CD8+ T cell-related ferroptosis genes *via* GO and KEGG Analysis; construction of protein-protein interaction (PPI) network analysis based on CD8+ T cell-related ferroptosis genes

**Figure 3:**
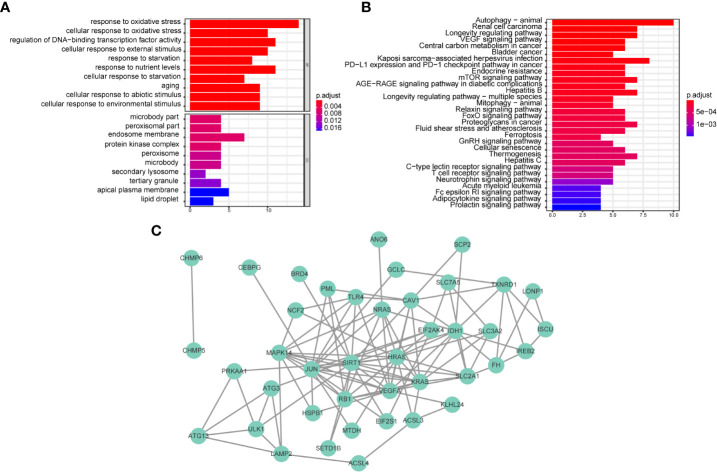
Biological functional and pathway enrichment analysis of CD8+ T cell-related ferroptosis genes. **(A, B)** GO and KEGG analysis on CD8+ T cell-related ferroptosis genes. **(C)** Network of co-expressed CD8+ T cell-related ferroptosis genes visualized by Cytoscape.

We further investigated the differences in biological processes and pathways in 46 ferroptosis-related differentially expressed genes (DEGs) between CD8+ ^high^ and CD8+ ^low^ T cell groups (**Figure 3A**). Pathways related to ‘response to oxidative stress’, ‘cellular response to oxidative stress’, ‘protein kinase complex’, and ‘secondary lysosome’ etc. were enriched by GO analysis. In the process of KEGG analysis, pathways related to ‘acute myeloid leukemia’, ‘PD-L1 expression’ and ‘PD-1 checkpoint pathway in cancer’ etc. were enriched (**Figure 3B**). As shown in **Figure 3C**, we constructed a protein-protein interaction (PPI) network using Cytoscape based on the 46 CD8+ T cell-related ferroptosis genes.

### Defining a prognostic ferroptosis-related gene signature for CD8+ T cells in AML

**Figure 4:**
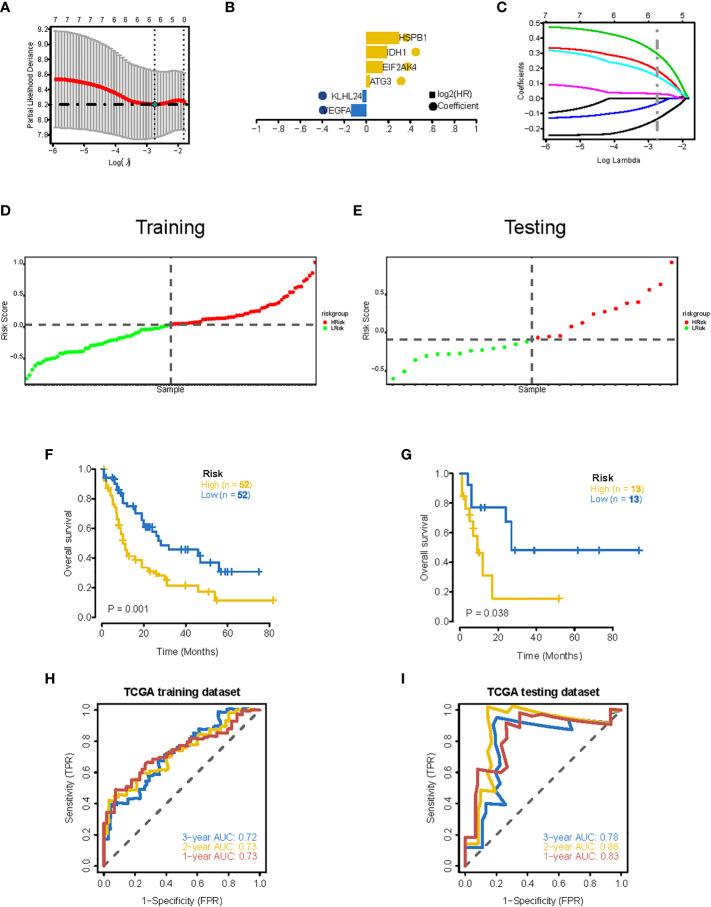
Construction of 6-gene prognostic signature. **(A)** Determination of the minimum lambda value with ten-fold cross validation of tuning parameter selection in the LASSO model. **(B)** Distribution of LASSO coefficients for prognosis-related genes and the gene combinations at the minimum lambda value. **(C)** Coefficients of Lasso genes. **(D, E)** The distribution of the risk scores in the training and testing datasets. **(F, G)** Survival curves of TCGA training dataset and testing dataset. **(H, I)** ROC curves of TCGA training dataset and testing dataset (AUC values at 1, 2 and 3 years in both the training and testing datasets were greater than 0.7).

To establish a ferroptosis-related gene expression signature, we integrated clinical information from TCGA-LAML cohort and randomly divided these patients into training and validation sets using a 4:1 ratio. By jointly using univariate Cox regression and LASSO regression analysis (**Figures 4A, B**), we generated an optimal six-gene prognostic signature (Gene6): Risk Score = VEGFA x (-0.136195304538939) + KLHL24 x (-0.0344312172907215) + ATG3 x 0.0306216446230082 + EIF2AK4 x 0.146417351223169 + IDH1 x 0.186205021011148 + HSPB1 x 0.296526705974414 (**Figure 4C**). The distribution of risk score was then analyzed (**Figures 4D, E**), which demonstrates that prognostic model has the ability to distinguish high- and low-risk groups of AML patients. Based on the median risk score, patients were classified into high-risk and low-risk groups, and Kaplan-Meier curves were utilized to analyze the data. In both the validation and training sets, OS was considerably lower in the high-risk group (**Figures 4F, G**). Additionally, the AUC values at 1, 2, and 3 years were greater than 0.7 in both the testing and training datasets, as shown by the ROC curves, suggesting that the model had robust predictive power (**Figures 4H, I**).

### Independent validation of the prognostic six-gene signature

**Figure 5:**
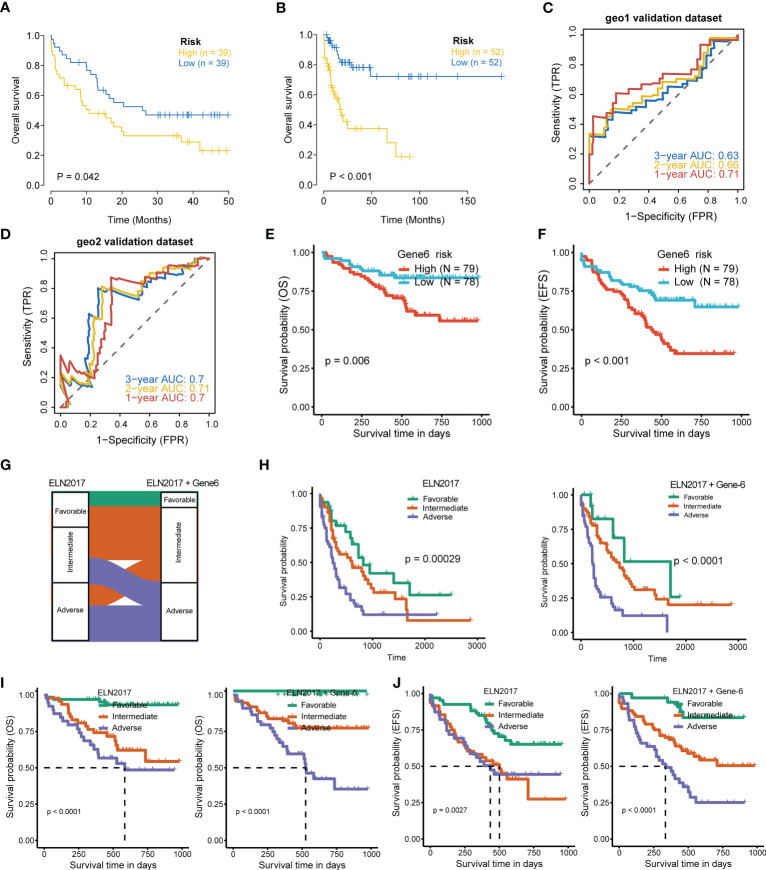
Validation of 6-gene prognostic signature. **(A, B)** Kaplan-Meier curves for OS of high- and low-risk groups in validation datasets (GSE12417 dataset, geo1; GSE71014 dataset, geo2). **(C, D)** ROC curves of the risk score in geo1 and geo2 validation gene set (AUC values > 0.6 at 1, 2, and 3 years in the geo1 validation dataset; AUC values > 0.7 at 1, 2, and 3 years in the geo2 validation dataset). **(E, F)** OS and EFS of high- and low-risk groups in RJAML cohort. **(G)** Improvement of the ELN2017 classification incorporating with Gene6 signature. **(H)** Comparison of OS segregation between different risk classifications in ELN2017 vs ELN2017 plus Gene6 at TCGA-LAML cohort. **(I, J)** Comparison of OS and EFS segregation between different risk classifications in ELN2017 vs ELN2017 plus Gene6 at RJAML cohort.

To further evaluate the association of the six-gene signature with patient survival, we obtained the gene expression and clinical data from the GSE12417 (GPL570) (24) and GSE71014 (25) independent patient cohorts. Compared with low-risk scores, patients with high-risk scores had significantly poorer OS than those, according to Kaplan-Meier analysis (**Figures 5A, B**). ROC analysis also showed that the six-gene model had high accuracy in predicting patient prognoses (AUC values > 0.6 at 1, 2, and 3 years in the GSE12417 dataset; AUC values > 0.7 at 1, 2, and 3 years in the GSE71014 dataset) (**Figures 5C, D**). Similarly, differences in survival between the high- and low-risk groups remained significant in RJAML cohort, indicated by shorter OS and event-free survival (EFS), in high-risk patients (**Figures 5E, F**). Collectively, these results demonstrated the prognostic power of the six-gene signature. Incorporation of the six-gene signature into the ELN2017 scheme resulted in generating three new risk groups, as follows: patients with Gene6^low-risk^/ELN-favorable were re-classified into the favorable-risk group, whereas Gene6^high-risk^/ELN-favorable, Gene6^low-risk^/ELN-intermediate and Gene6^low-risk^/ELN-adverse patients were re-classified as the intermediate-risk group, and Gene6^high-risk^/ELN-intermediate patients were re-assigned to the adverse-risk group (**Figure 5G**). Based on the median OS value, these resulting ELN2017 plus Gene6 scores contributed to improved risk segregation and substantially refined ELN2017 classification in TCGA-LAML cohort, evidenced by the more significant *p*-value (**Figure 5H**). Similar results were obtained when these analyses were performed independently in the RJAML cohort, regardless of OS or EFS (**Figures 5I, J**). Taken together, these results demonstrated that the Gene6 signature could improve the prognostic efficacy of ELN2017 classification. In addition, we analyzed the association of 6 signature genes expression with the infiltration of 22 major immune cell types and the response to immunotherapy in AML (**Figures 2S**, **3S**).

### Gene mutation, drug sensitivity, GSEA, and GSVA analysis between high-risk group and low-risk group classified by CD8+ T cell-related ferroptosis prognostic signature in AML

**Figure 6:**
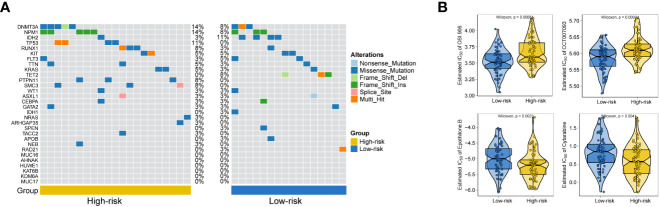
Gene mutation analysis and drug sensitive prediction in AML patients. **(A)** Oncoplots showing AML-related gene mutation frequencies among high-risk group and low-risk group in AML patients. **(B)** Violinplots showing the mean differences in estimated IC50 values of 4 representative drugs (OSI.906, CCT007093, Epothilone.B, Cytarabine) between the two risk groups.

**Figure 7:**
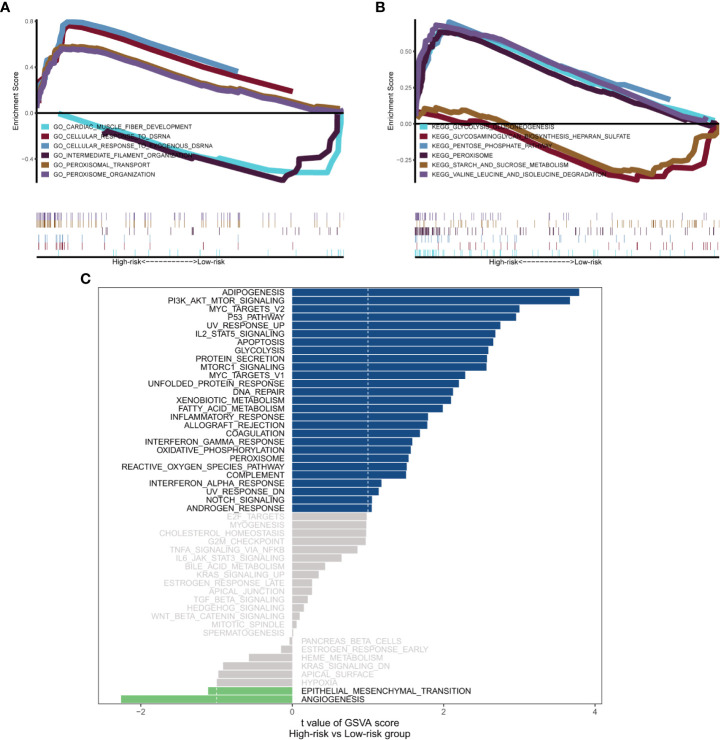
Biological phenotypes associated with Gene6 risk score. **(A, B)** GSEA results of Gene6 risk score in GO and KEGG. **(C)** GSVA result of Gene6 risk score.

General information on AML-related gene mutations in high- versus low-risk groups is shown in **Figure 6A**. DNMT3A (14%), NPM1 (14%), TP53 (11%), RUNX1 (8%) and PTPN11 (8%) rank top five on gene mutation frequencies in high-risk group. The top five genes in the low-risk group with the highest mutation frequencies were IDH2 (11%), TET2 (8%), NPM1 (8%), DNMT3A (8%) and FLT3 (5%). We contrasted the estimated IC50 levels of drugs in AMLs from two groups. **Figure 6B** shows four representative drugs. Epothilone.B and Cytarabine were identified to be potential treatment options for patients in high-risk group. Conversely, OSI.906, CCT007093 turn out not ideal drugs for AMLs stratified to high-risk group by Gene6. We then characterized the biological phenotypes associated with each Gene6 risk score through gene set enrichment analysis (GSEA). Indicated by GSEA, upregulated genes were enriched in pathways related to ‘cellular response to exogenous dsRNA’ and ‘valine, leucine, and isoleucine degradation’, while down-regulated genes were involved in pathways related to ‘intermediate filament organization’, ‘glycosaminoglycan biosynthesis - heparan sulfate’ in the high-risk patient group (**Figures 7A, B**). Further gene set variation analysis (GSVA) showed that altered pathways in the high-risk patient group were mainly involved in ‘adipogenesis’, ‘PI3K/AKT/MTOR signaling’, ‘IL2/STAT5 signaling’, ‘mTORC1 signaling’ and others (**Figure 7C**).

## Discussion

In the current work, we first quantified the fraction of various immune infiltrating cells in each patient sample with CIBERSORT (22) to divide the samples into CD8+ ^high^ and CD8+ ^low^ T cell groups by the median percentage of infiltrating CD8+ T cells. We then identified ferroptosis-related genes that were differentially expressed in CD8+ ^high^ and CD8+ ^low^ T cell groups. We then collected clinical information from each AML patient, identified AML-related signature genes, and used LASSO regression to obtain a panel of genes that provided optimal risk scores for subsequent analysis. To validate the accuracy of the model, we conducted ROC analysis using two external datasets downloaded from GEO database and RNA-seq data from 157 bone marrow (BM) samples aspirated from 157 *de novo* AML patients diagnosed at Ruijin Hospital. It is noteworthy that combining our Gene6 model can improve the accuracy of ELN2017 in classifying the prognosis of AML patients (26).

The prognostic model proposed in this study consists of 6 CD8+ T cell infiltration-related ferroptosis genes (*HSPB1*, *IDH1*, *EIF2AK4*, *ATG3*, *KLHL24*, *VEGFA*). Consistent with the strong connection between this prognostic model and CD8+ T cell immunity, several previous reports revealed that HSPB1 plays a part in the control of CD8+ T cell (27–29). On the other hand, HSPB1 phosphorylation protects tumor cells from ferroptosis by reducing lipid ROS production mediated by iron (30). Glutathione oxidase 4 (GPX4) is a key enzyme in the removing lipid reactive oxygen species (ROS) and ferroptosis. In fact, prior to the recent publication of direct evidence that CD8 can function in cancer *via* ferroptosis (20), numerous previous works unveiled that ferroptosis enhances anti-cancer immunity by boosting cancer immunosurveillance and deciding the fate of CD8+ T cells *via* glutathione oxidase 4 (GPX4), which is a key enzyme in the removing lipid reactive oxygen species (ROS) and ferroptosis (31, 32). IDH1 mutation affects the prognosis of AMLs (33) through regulating the protein level of GPX4 and so has a connection with CD8+ T cell either (34). EIF2AK4 (or GCN2) has been found to affect the survival of tumor patients by suppressing cancer immunology (35, 36). In addition, EIF2AK4 mediates anergy induction and proliferative arrest in T cell through detecting and responding to the immunoregulatory signal generated by Indoleamine 2,3 dioxygenase (IDO) (37). This may support the finding that GCN2 is positively associated with risk of AMLs in our prognostic model based on CD8+ T cell-associated ferroptosis gene. Besides, demonstrated by PPI network of CD8+ T cell-associated ferroptosis genes in **Figure 3C**, both IDH1 and EIF2AK4 are co-expressed with *Solute Carrier Family 7 Member 5* (SLC7A5), a type of system L transporter. Previous research revealed that T cells require SLC7A5 for methionine uptake even though methionine can also be produced *de novo* in mammalian cells as well (38, 39). Hence in combination with the previous research, the co-expression result not only reflects the fact that the two genes in Gene6 (IDH1 and EIF2AK4) are associated with CD8+ T cell but also indicates that this association may be mediated by SLC7A5. Jia et al. demonstrated that survival of naïve CD8+ T cell is defective in ATG3-deficient T lymphocytes, which indicated ATG3 is associated with CD8+ T cell (40). Meanwhile, ferroptosis also has been known as a type of cell death depending on autophagy in which ATG3 is a key player (41, 42). In a study conducted by Altman et al, ATG3 deficiency prevented BCR-abl-dependent leukemia by blocking the autophagic pathway (43). KLHL24 found in acute myeloid leukemia as an autophagy-related gene to inform prognostic assessment (38, 44). Despite the lack of previous evidence showed that KLHL24 directly correlated with CD8+ T cell, we speculate KLHL24 indirectly associated with CD8+ T cell *via* autophagy, as autophagy act a pivotal part in the cellular and metabolic reprogramming progresses of CD8+ T cell (45). VEGFA, has been widely recognized as a pro-angiogenic factor in vertebrates and a promoter of tumor progression for decades (46). However, in a research of Palazon et al., they found deletion of VEGFA in CD8+ T cell enhanced tumor growth, which was explained by the VEGF-deficient CTLs’ intrinsic defect in acquisition of effector phenotypes (47). Furthermore, in one of the latest studies, researchers found that the absence of CDS2 enhanced the level of VEGFA secreted by the tumor, thereby trapping the tumor in a condition of VEGFA-induced vascular regression, leading to inhibition of tumor growth, which may result in activation of ferroptosis-related pathways (48). Consistent with above mentioned reports, these 6 genes in our model are all directly or indirectly associated with both CD8+ T cell and ferroptosis in cancer and jointly affect the prognosis of AMLs.

As shown by the analysis of AML-related gene mutation between high and low-risk groups (**Figure 6A**), the adverse-risk gene mutations such as TP53, SMC3 and PTPN11 mutations (26, 49, 50) have obviously higher frequency in high-risk group. This study also excavated altered biological phenotypes including ‘cellular response to exogenous dsRNA’, ‘PI3K/AKT/MTOR signaling’ and so on in high- versus low-risk patient group by GSEA and GSVA. Consistently, exogenous dsRNA participates in driving Type I IFN induction *via* targeting RNA-sensing PRR pathways, including RIG-I/MDA5-IPS-1, TLR7-MyD88 and TLR3-TICAM-1 in dendritic cell subset, so as to evoke and amplify CD8+ T cell anti-tumor immunity (51). In contrast to subcutaneously grown leukemic tumors, diffused leukemia is known for its failure to produce type I IFN, which prevents it from generating cellular antitumor immunity (52, 53). FLT3-ITD was previously reported to synergistically activate the mTORC1/S6K/4EBP1 pathway through the PI3K/AKT and STAT5/PIM pathways to enhance eIF4F complex formation and promote the proliferation and survival of tumor cells, while FLT3-ITD is the most common tyrosine kinase mutation associated with poor prognosis in AML (54). The association of other pathways with prognosis was also illustrated in previous reports (55–57). These findings suggested that perturbation of these signaling pathways may underpin the survival differences between the high-risk and low-risk patient groups.

In general, this study was the first to establish a prognostic model of AML based on CD8^+^ T cell-associated ferroptosis genes that exhibited certain guiding effects in predicting OS and EFS in patients with AML and further optimize the classification of ELN2017 scheme, which is proved by multiple data sets, especially our patient sample data. In order to reduce the limitations of the study, we hope that the utility of the model constructed will also be validated in the future with larger scale of clinical samples and studies. Additionally, the complex mechanisms underlying the prognostic function of 6 CD8+ T cell-related ferroptosis genes in AML need being further investigated in the following work.

## Data availability statement

The datasets presented in this study can be found in online repositories. The names of the repository/repositories and accession number(s) can be found below: Gene Expression Omnibus (GEO) *via* GSE201492.

## Ethics statement

The study was approved by the Ethics Review Committee of Ruijin Hospital affiliated to Shanghai Jiao Tong University School of Medicine. The patients/participants provided their written informed consent to participate in this study.

## Author contributions

JL, GJ, XL and KX designed the study. GJ, PJ, JS and XX drafted the paper. YZ, GJ, RL collected the samples. GJ, XX and PJ integrated and analyzed the data. GJ, PJ, XX and KX edited and revised the paper. All authors contributed to the article and approved the submitted version.
