# Supplementary figures and images for: Identification and validation of a novel CD8+ T cell-associated prognostic model based on ferroptosis in acute myeloid leukemia
Source: Front Immunol. 2023 Apr 17;14:1149513. doi: 10.3389/fimmu.2023.1149513 (PMC10150955; doi:10.3389/fimmu.2023.1149513)

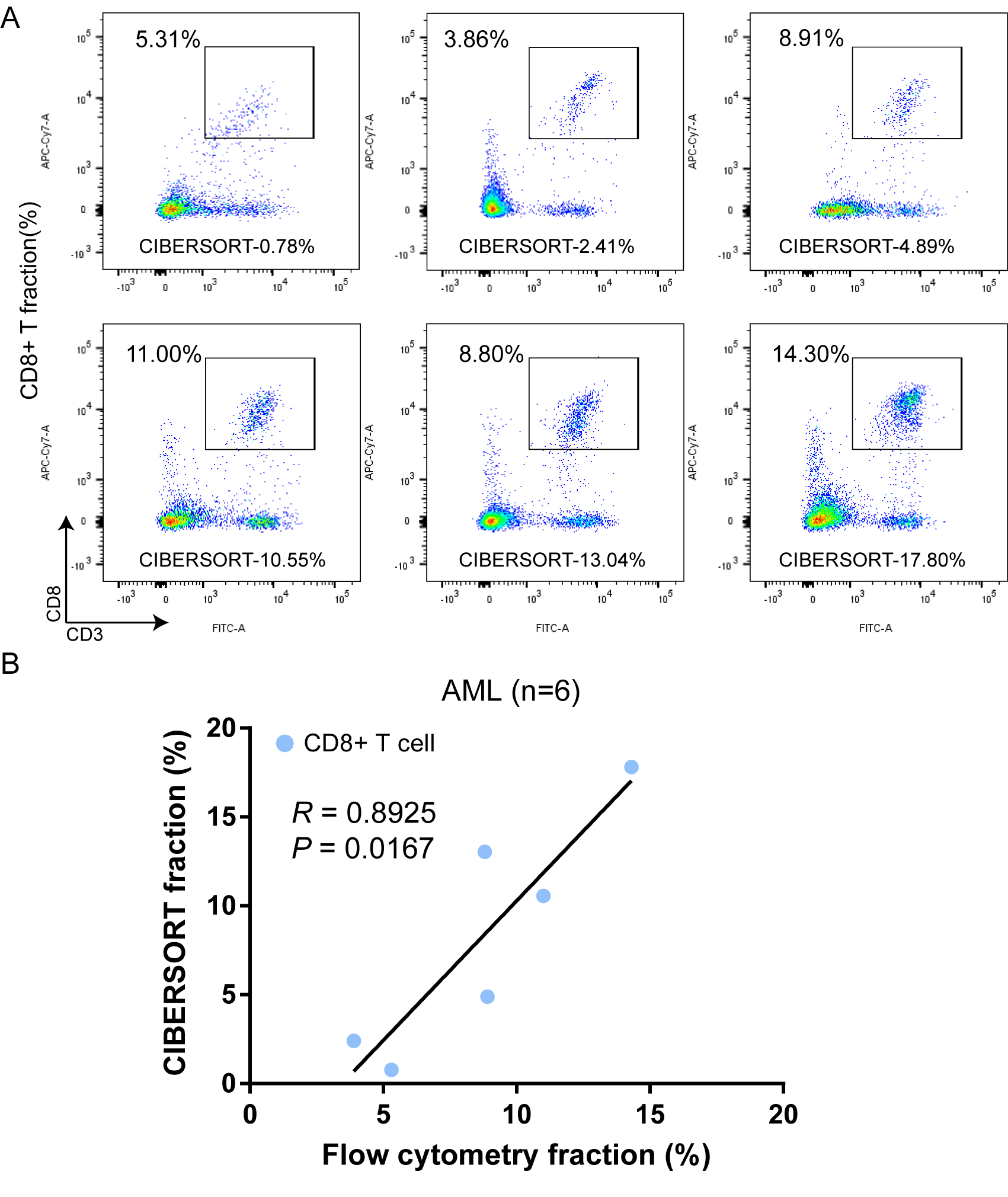

Supplement: Supplementary file 1 [file Image_1.tif]

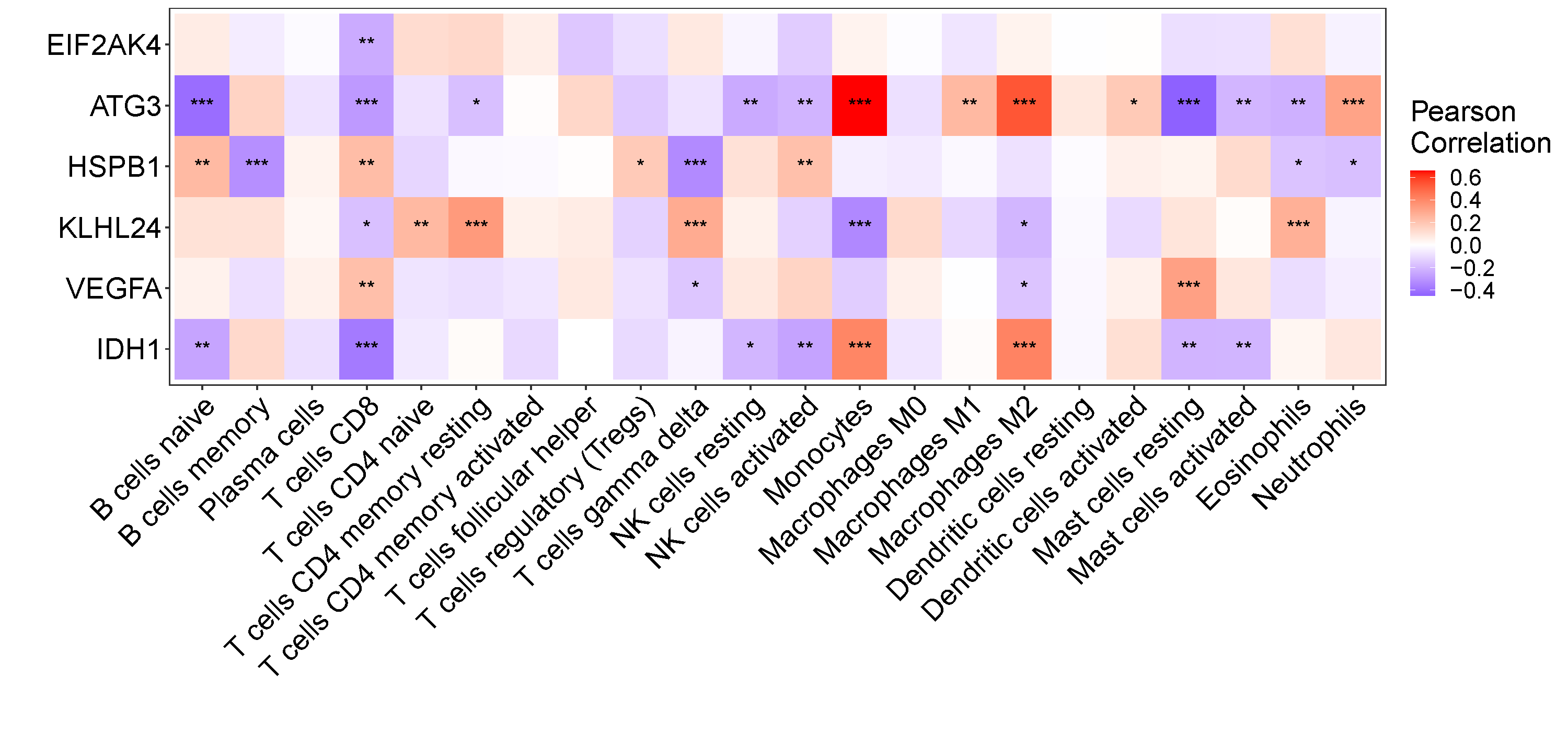

Supplement: Supplementary file 2 [file Image_2.tif]

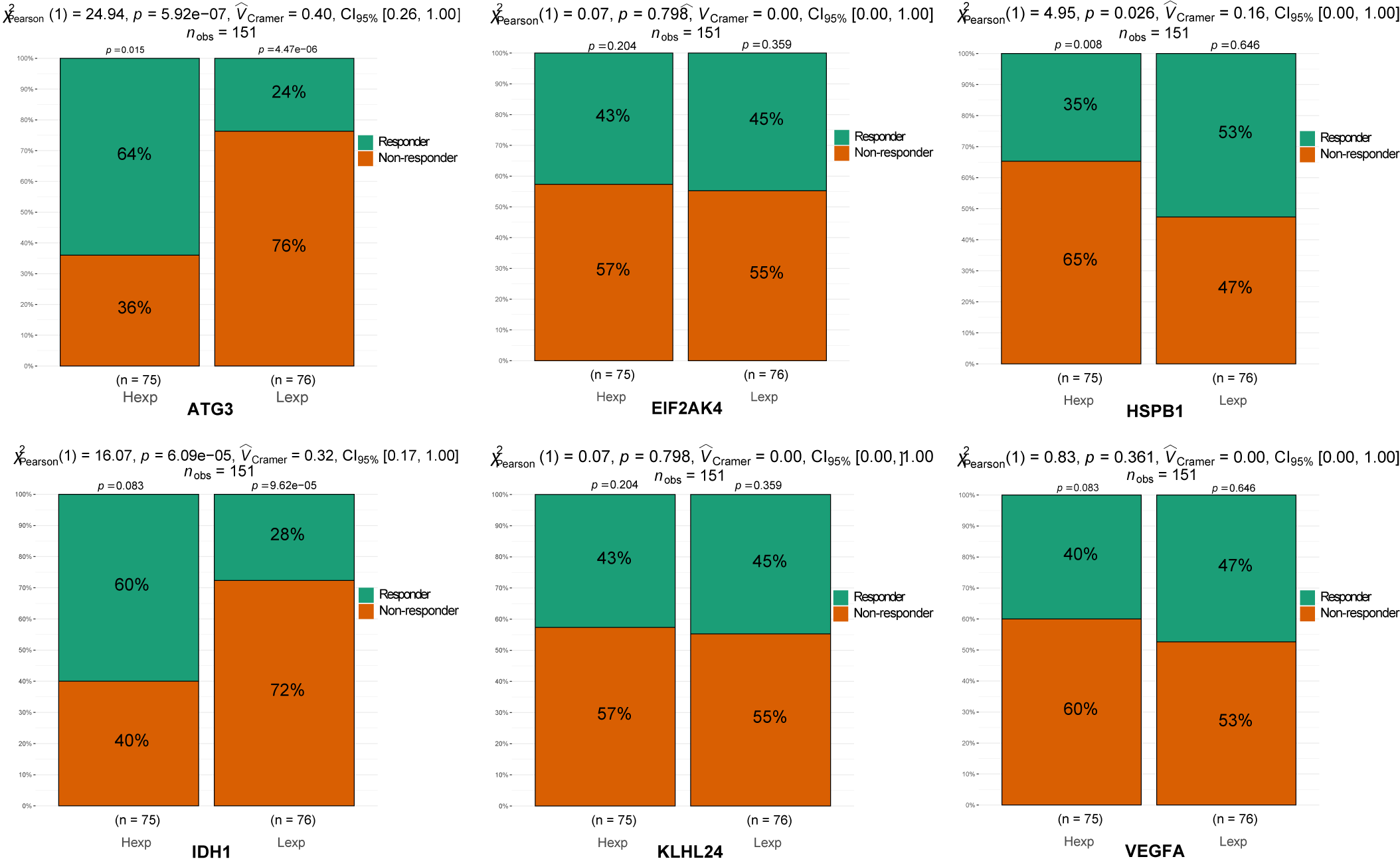

Supplement: Supplementary file 3 [file Image_3.tif]

Wilcoxon,  $p = 0.0018$

ACSL3 Expression

3.5  
3.0  
2.5  
2.0  
1.5

High

Low

High  
Low

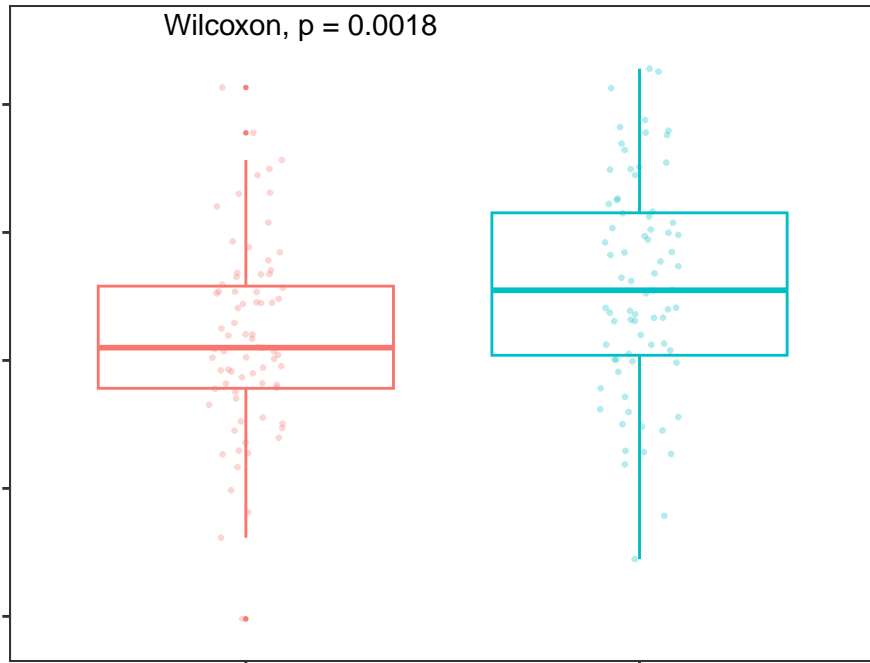

Supplement: Supplementary file 5 [file DataSheet_2.zip › expression/ACSL3.pdf]

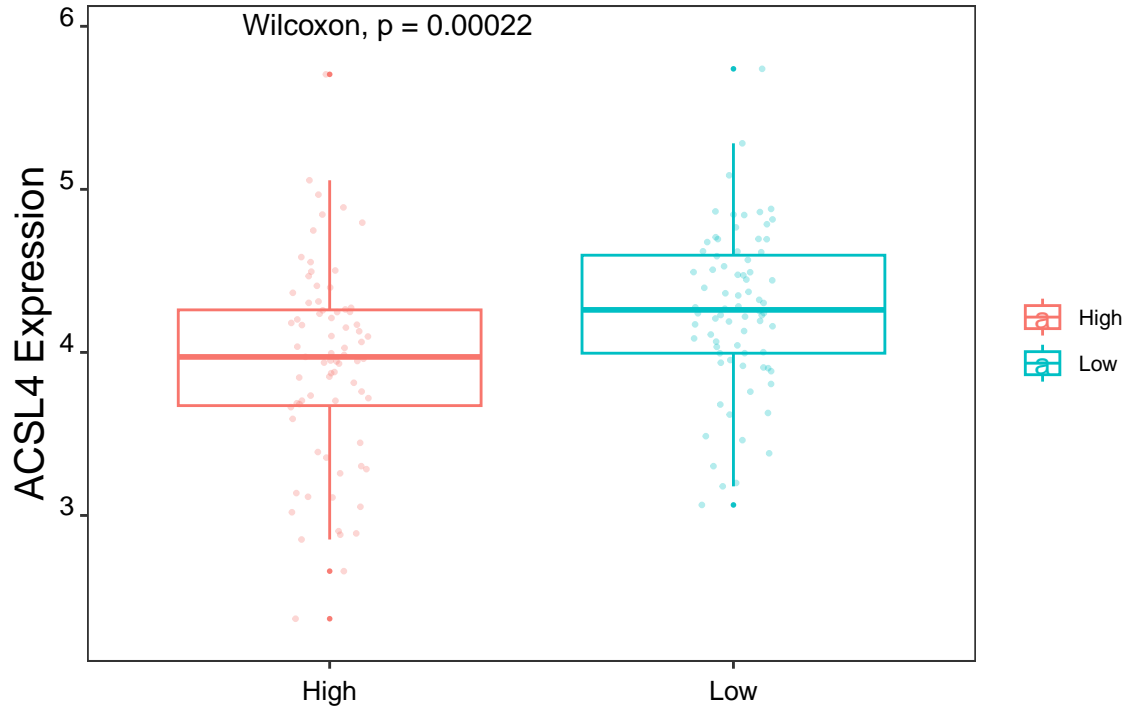

Supplement: Supplementary file 5 [file DataSheet_2.zip › expression/ACSL4.pdf]

ANO6 Expression

Wilcoxon,  $p = 0.00017$

5

4

3

2

High

Low

High  
Low

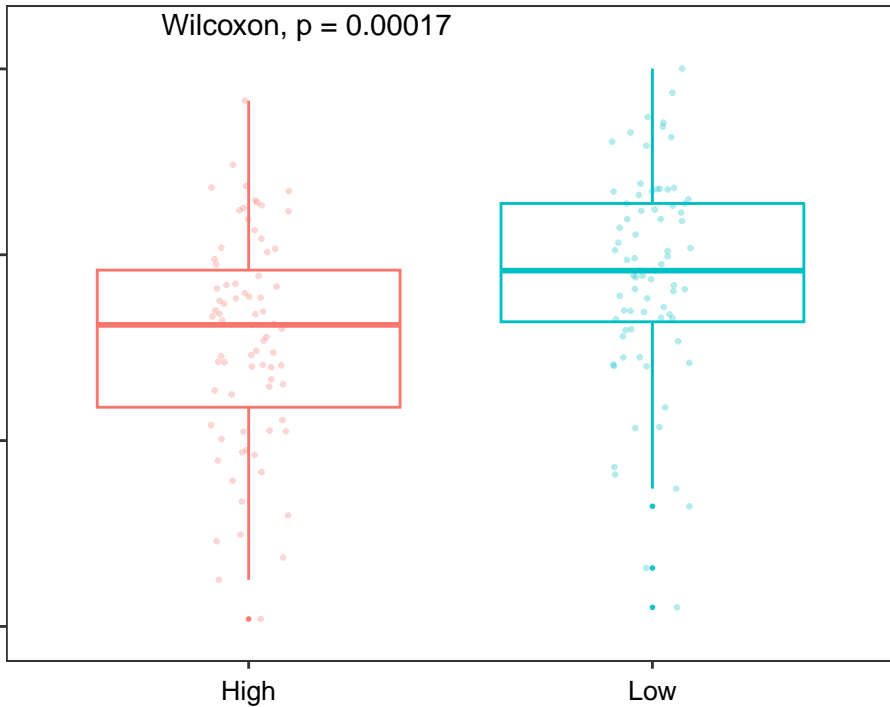

Supplement: Supplementary file 5 [file DataSheet_2.zip › expression/ANO6.pdf]

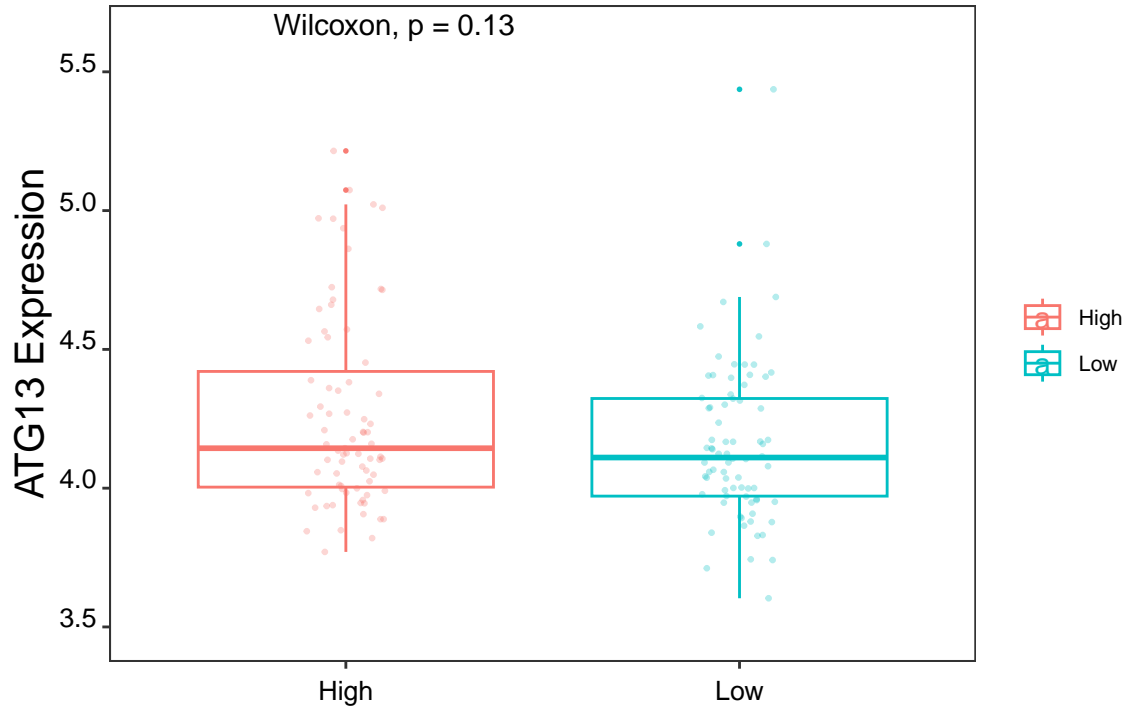

Supplement: Supplementary file 5 [file DataSheet_2.zip › expression/ATG13.pdf]

ATG3 Expression

Wilcoxon,  $p = 0.00042$

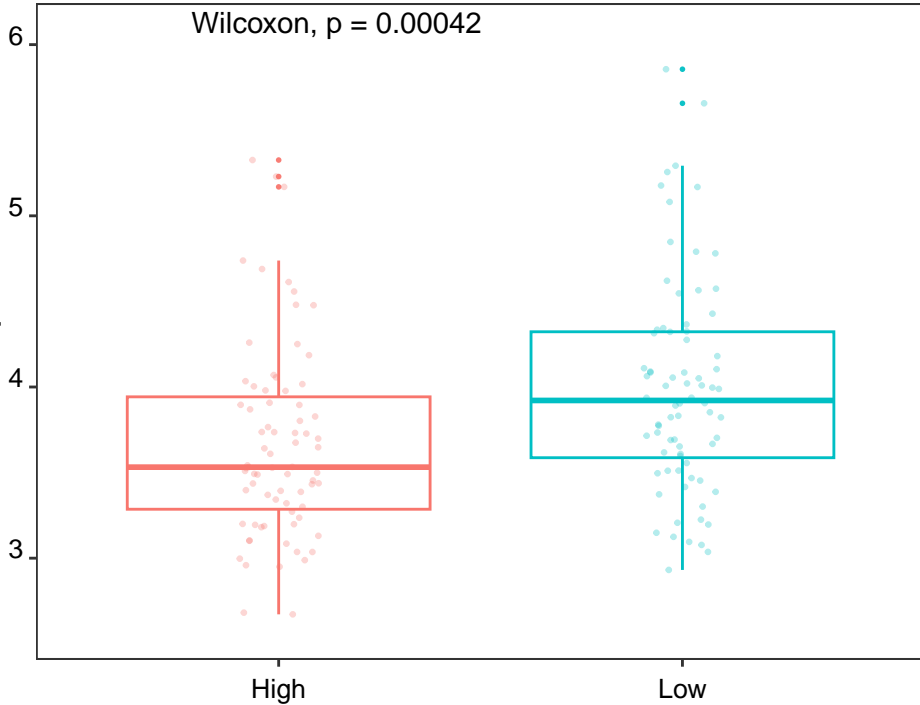

High  
Low

Supplement: Supplementary file 5 [file DataSheet_2.zip › expression/ATG3.pdf]

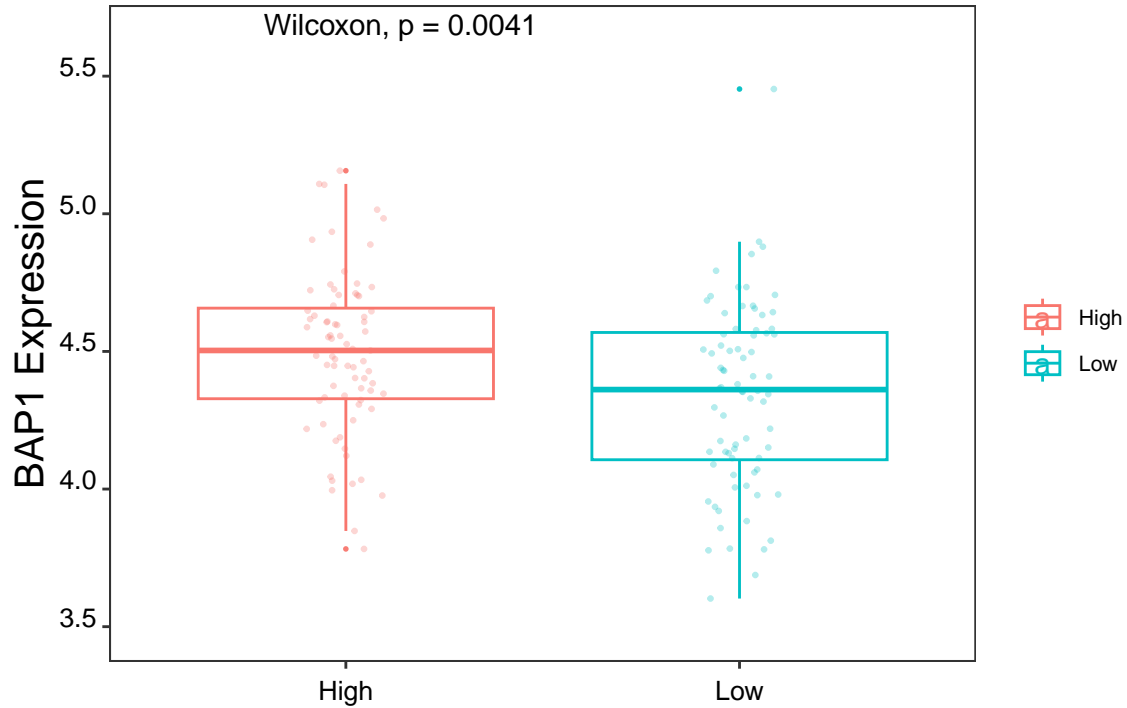

Supplement: Supplementary file 5 [file DataSheet_2.zip › expression/BAP1.pdf]

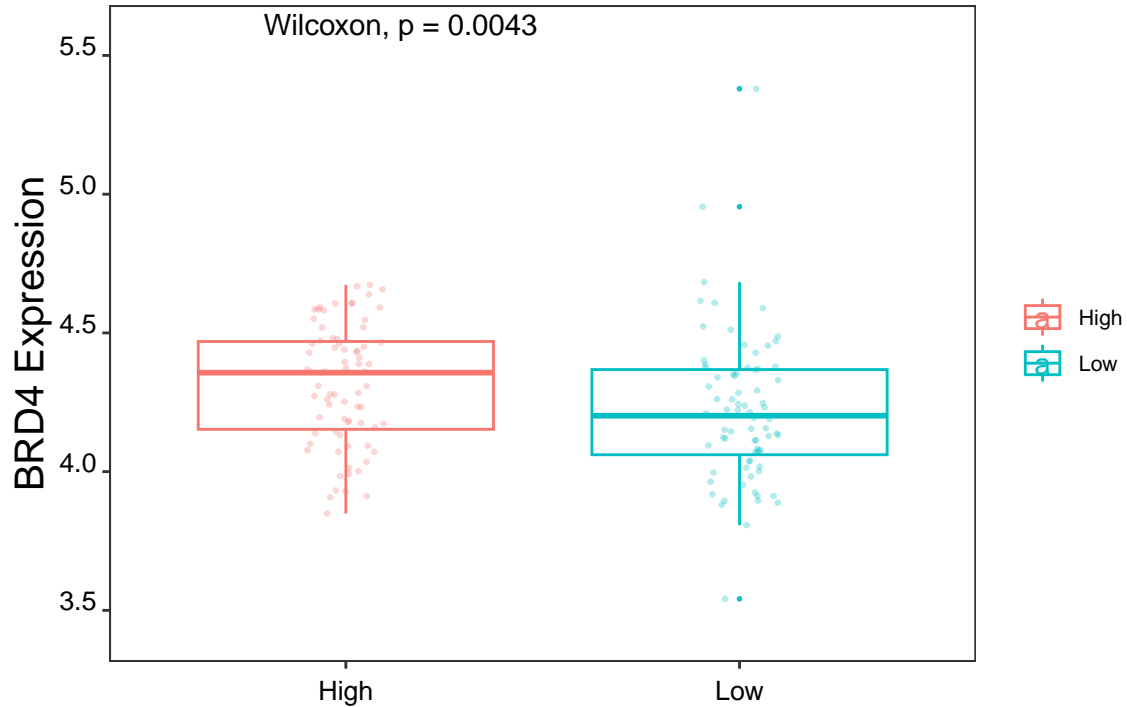

Supplement: Supplementary file 5 [file DataSheet_2.zip › expression/BRD4.pdf]

CAV1 Expression

Wilcoxon,  $p = 0.027$

6

4

2

0

High

Low

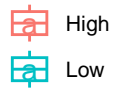

Supplement: Supplementary file 5 [file DataSheet_2.zip › expression/CAV1.pdf]

Wilcoxon,  $p = 0.0042$

CEBPG Expression

5

4

3

High

Low

High  
Low

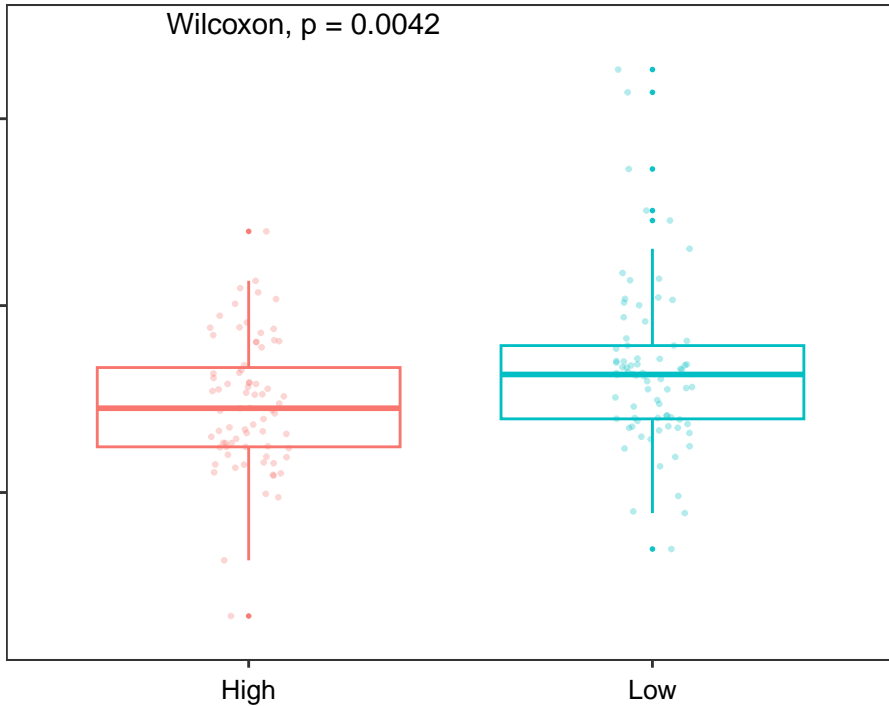

Supplement: Supplementary file 5 [file DataSheet_2.zip › expression/CEBPG.pdf]

CHMP5 Expression

Wilcoxon,  $p = 0.014$

5

4

3

High

Low

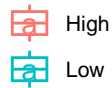

Supplement: Supplementary file 5 [file DataSheet_2.zip › expression/CHMP5.pdf]

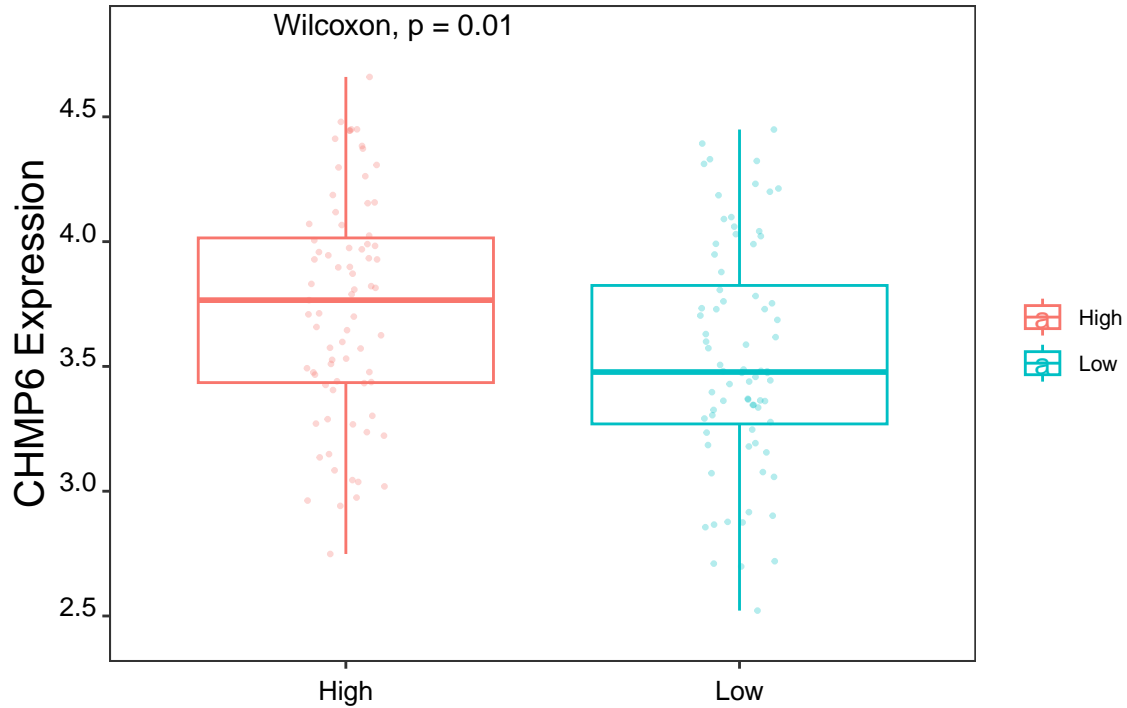

Supplement: Supplementary file 5 [file DataSheet_2.zip › expression/CHMP6.pdf]

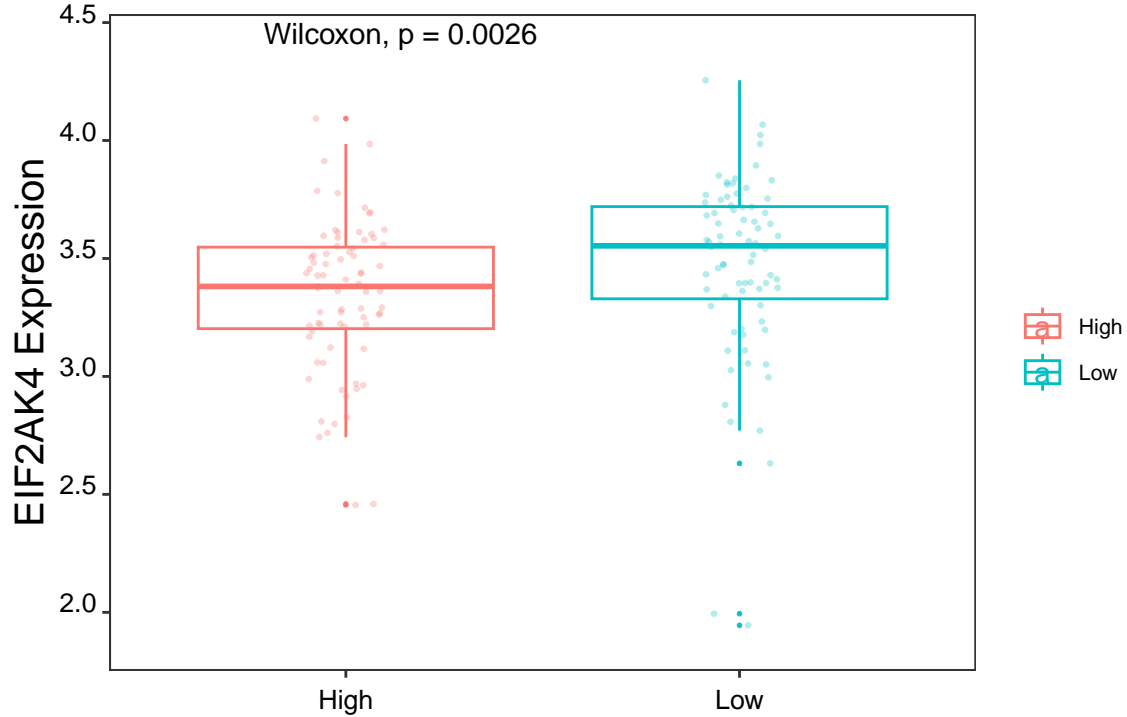

Supplement: Supplementary file 5 [file DataSheet_2.zip › expression/EIF2AK4.pdf]

EIF2S1 Expression

Wilcoxon,  $p = 3.8e-05$

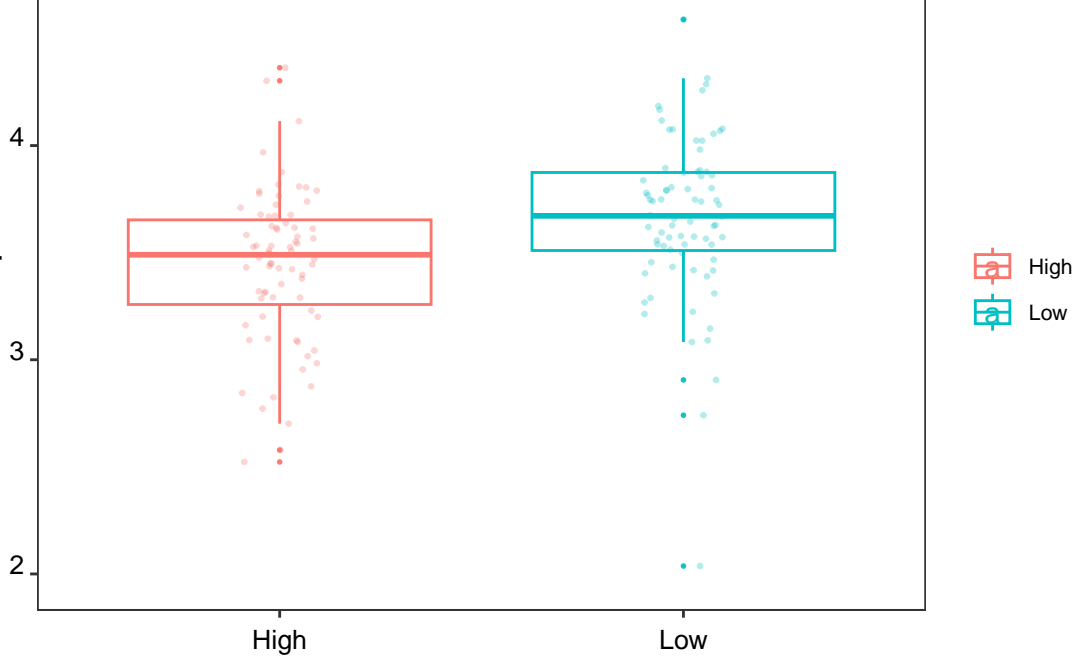

Supplement: Supplementary file 5 [file DataSheet_2.zip › expression/EIF2S1.pdf]

Wilcoxon,  $p = 0.0053$

FH Expression

5

4

3

High

Low

High  
Low

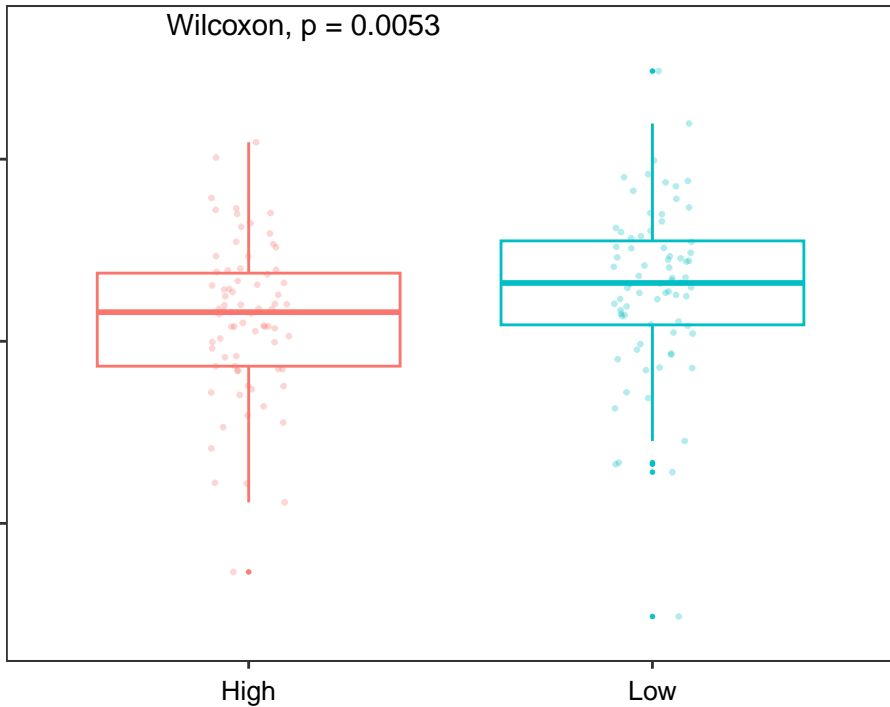

Supplement: Supplementary file 5 [file DataSheet_2.zip › expression/FH.pdf]

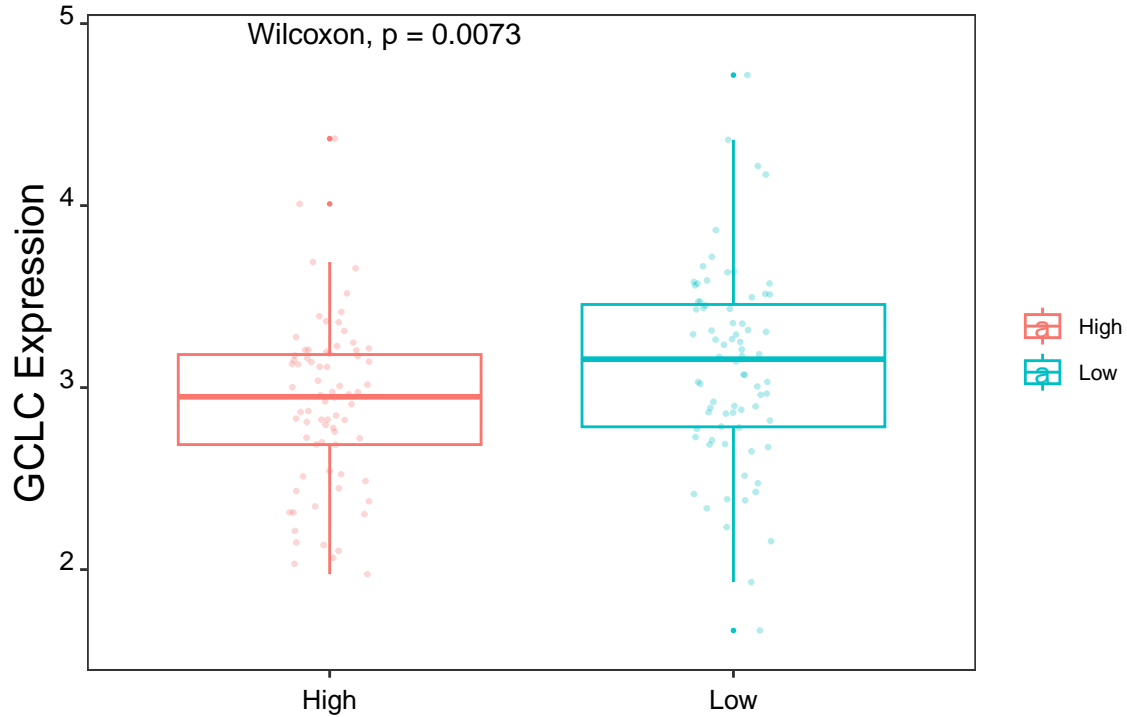

Supplement: Supplementary file 5 [file DataSheet_2.zip › expression/GCLC.pdf]

HBA1 Expression

Wilcoxon,  $p = 0.00019$

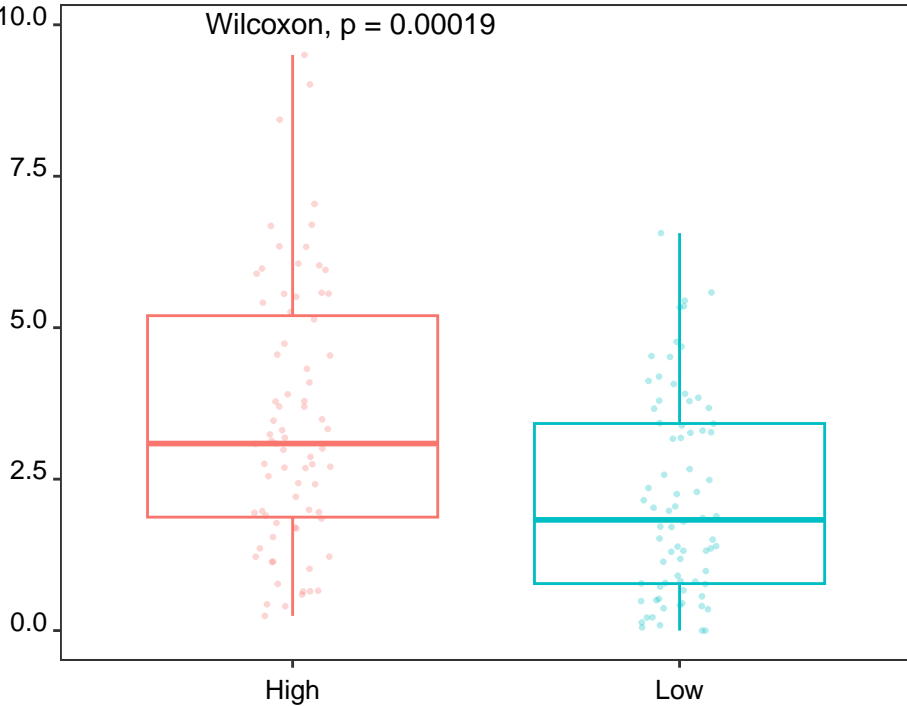

High  
Low

Supplement: Supplementary file 5 [file DataSheet_2.zip › expression/HBA1.pdf]

Wilcoxon,  $p = 0.0031$

HRAS Expression

4.0  
3.5  
3.0  
2.5  
2.0

High

Low

High  
Low

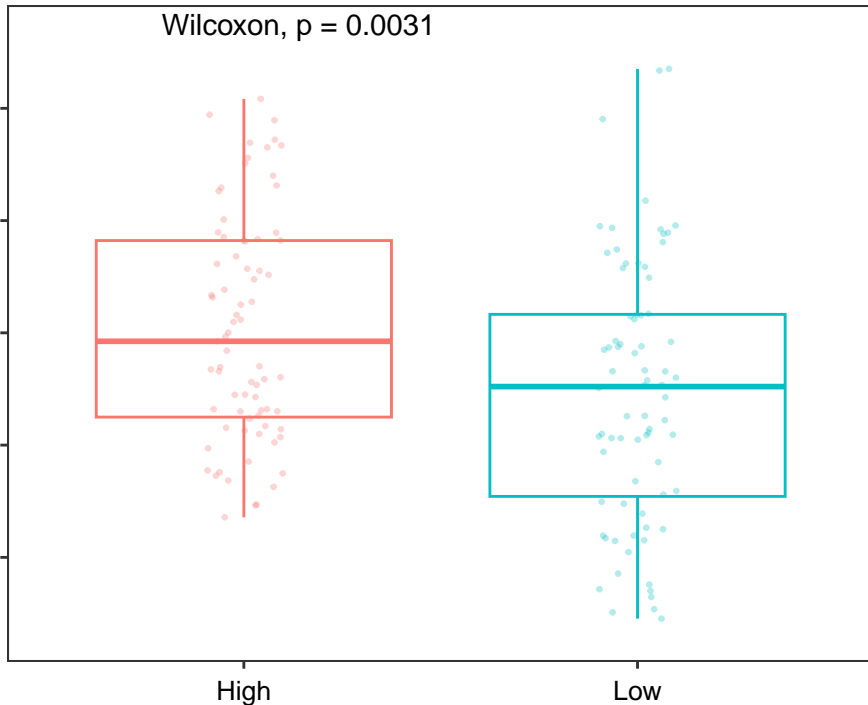

Supplement: Supplementary file 5 [file DataSheet_2.zip › expression/HRAS.pdf]

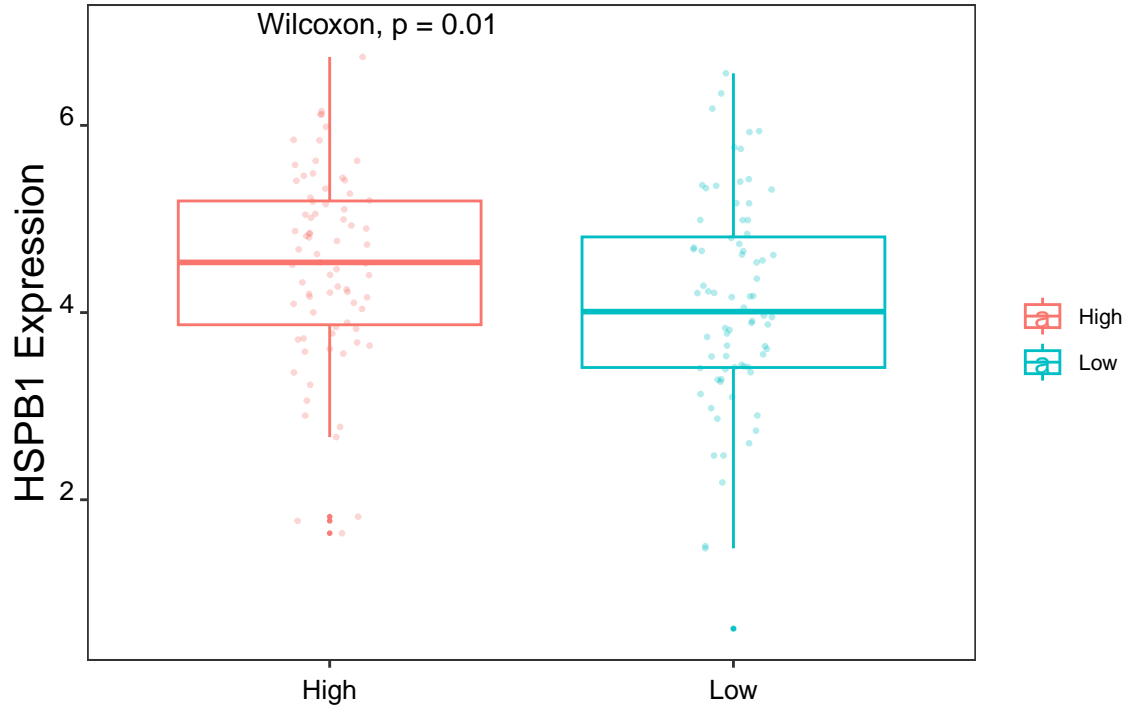

Supplement: Supplementary file 5 [file DataSheet_2.zip › expression/HSPB1.pdf]

Wilcoxon,  $p = 3.3\text{e-}05$

IDH1 Expression

6  
5  
4  
3

High

Low

High  
Low

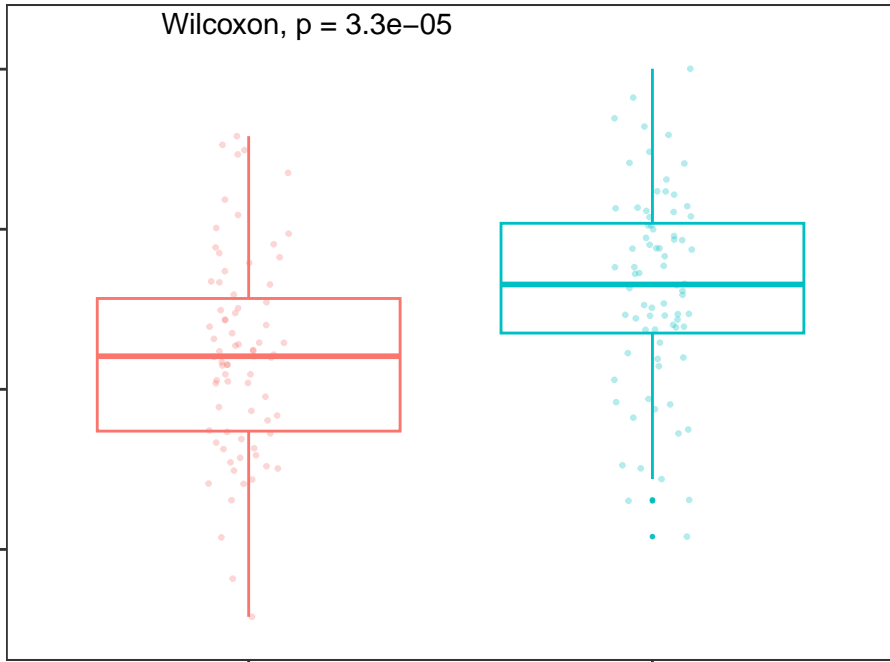

Supplement: Supplementary file 5 [file DataSheet_2.zip › expression/IDH1.pdf]

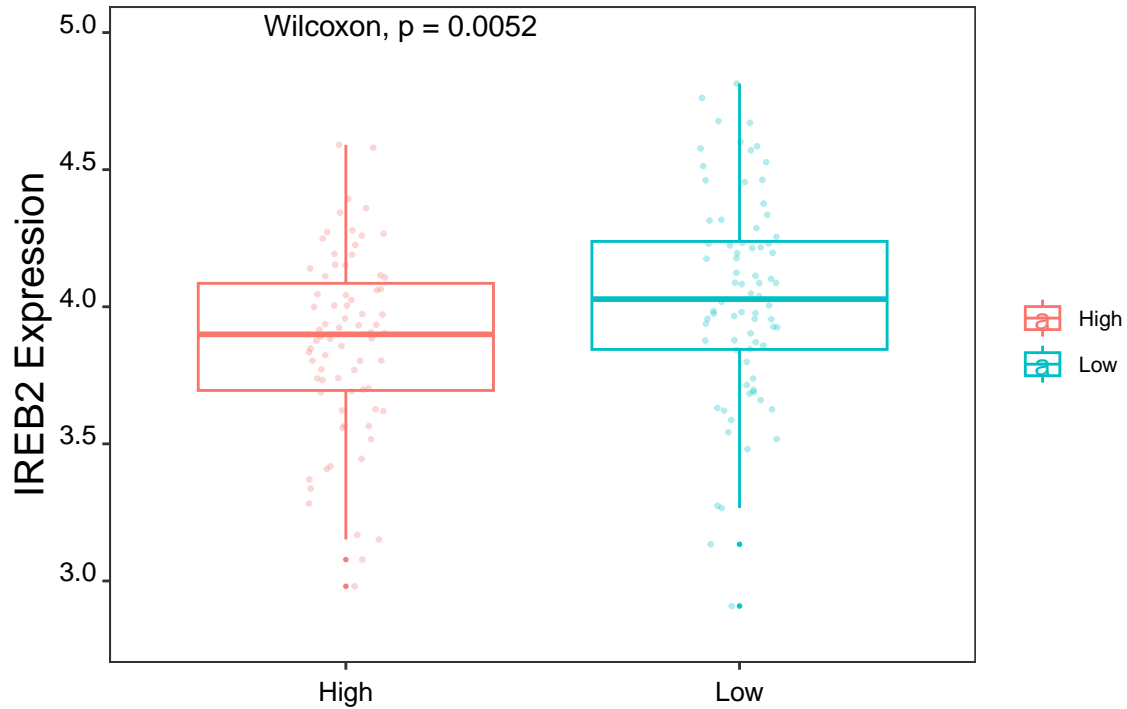

Supplement: Supplementary file 5 [file DataSheet_2.zip › expression/IREB2.pdf]

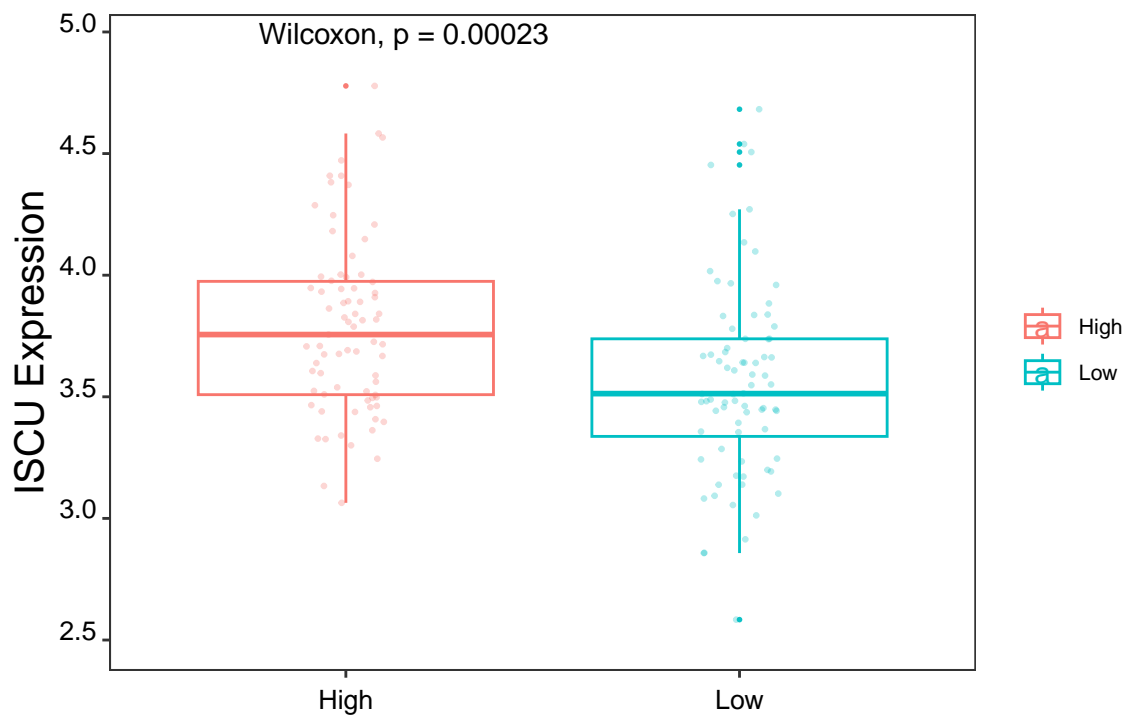

Supplement: Supplementary file 5 [file DataSheet_2.zip › expression/ISCU.pdf]

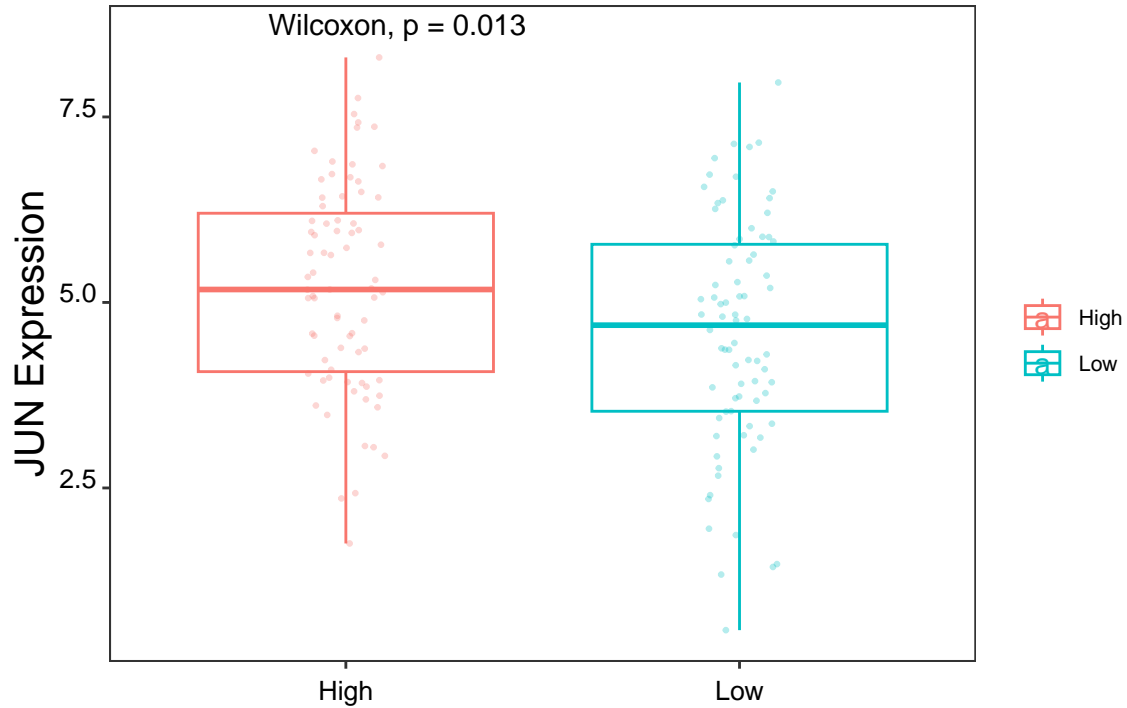

Supplement: Supplementary file 5 [file DataSheet_2.zip › expression/JUN.pdf]

Wilcoxon,  $p = 0.058$

KLHL24 Expression

5

4

3

High

Low

High  
Low

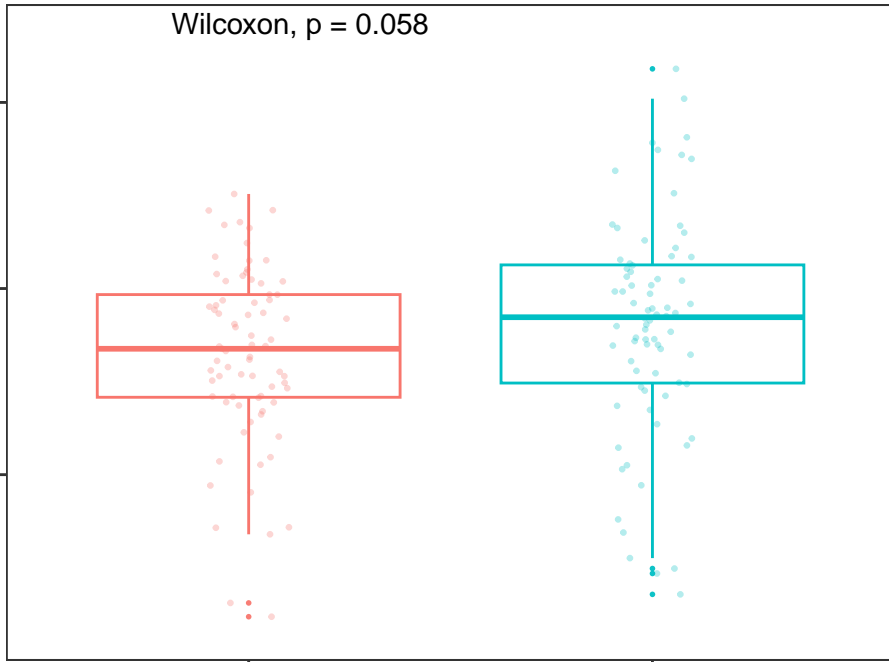

Supplement: Supplementary file 5 [file DataSheet_2.zip › expression/KLHL24.pdf]

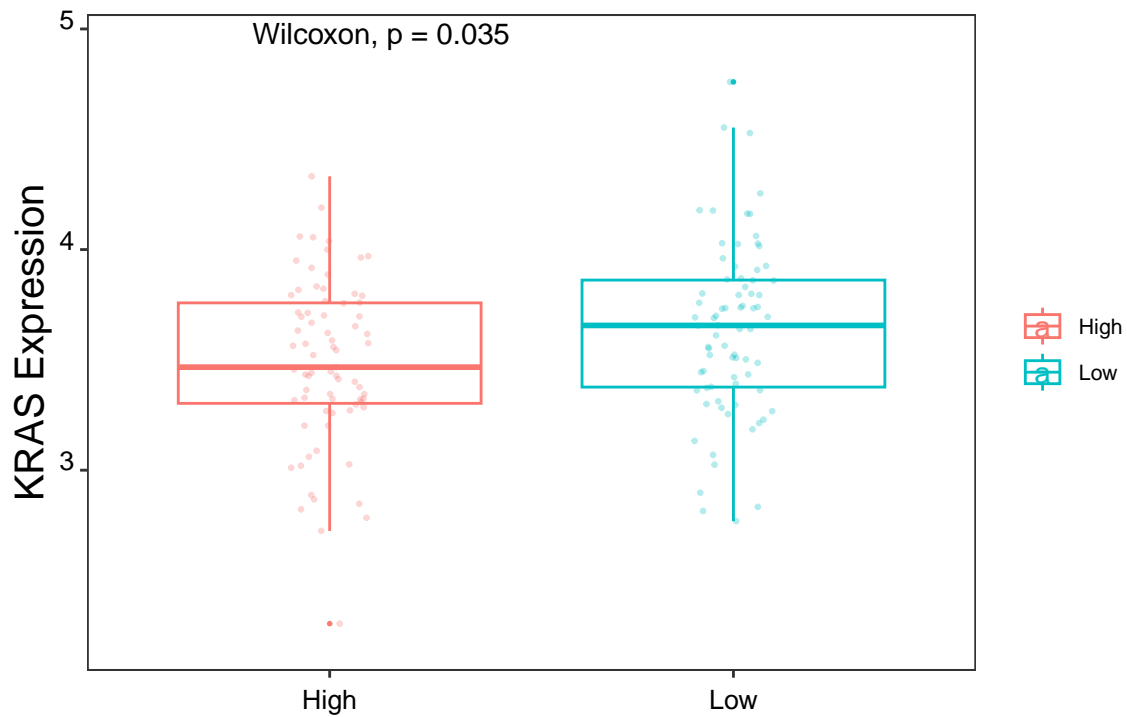

Supplement: Supplementary file 5 [file DataSheet_2.zip › expression/KRAS.pdf]

Wilcoxon,  $p = 0.0023$

LAMP2 Expression

4

5

6

High

Low

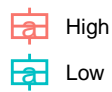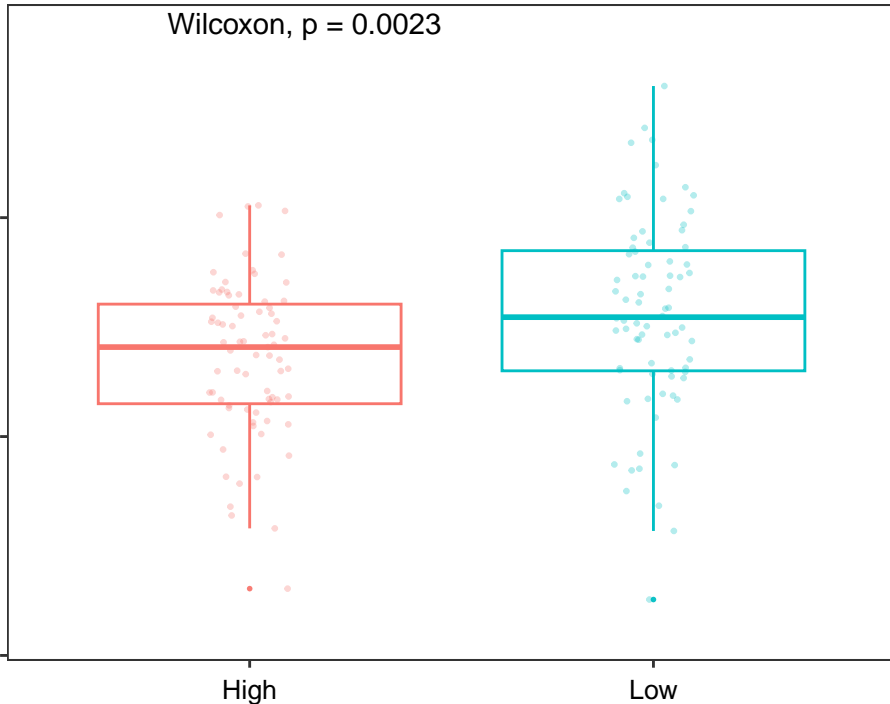

Supplement: Supplementary file 5 [file DataSheet_2.zip › expression/LAMP2.pdf]

LONP1 Expression

Wilcoxon,  $p = 0.00032$

5

4

3

High

Low

High  
Low

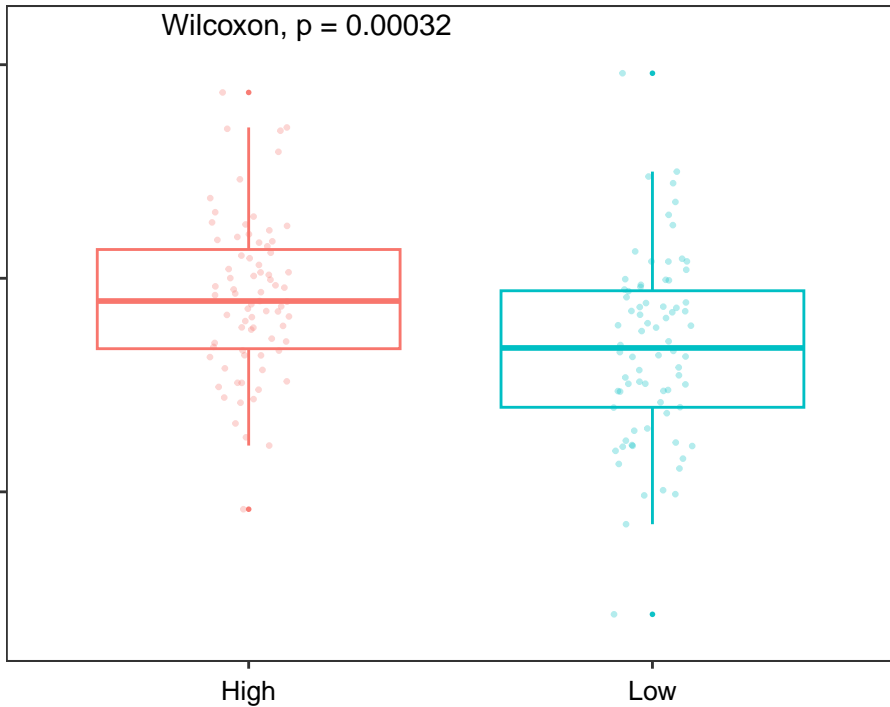

Supplement: Supplementary file 5 [file DataSheet_2.zip › expression/LONP1.pdf]

MAPK14 Expression

Wilcoxon,  $p = 0.0033$

6

5

4

High

Low

High  
Low

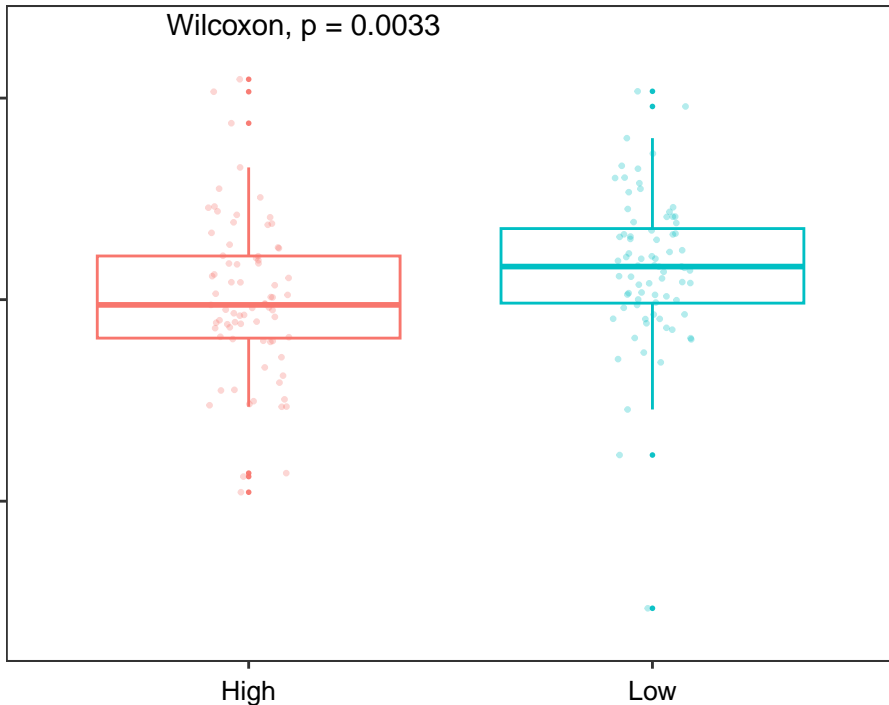

Supplement: Supplementary file 5 [file DataSheet_2.zip › expression/MAPK14.pdf]

MTDH Expression

Wilcoxon,  $p = 3e-04$

7

6

5

4

High

Low

High  
Low

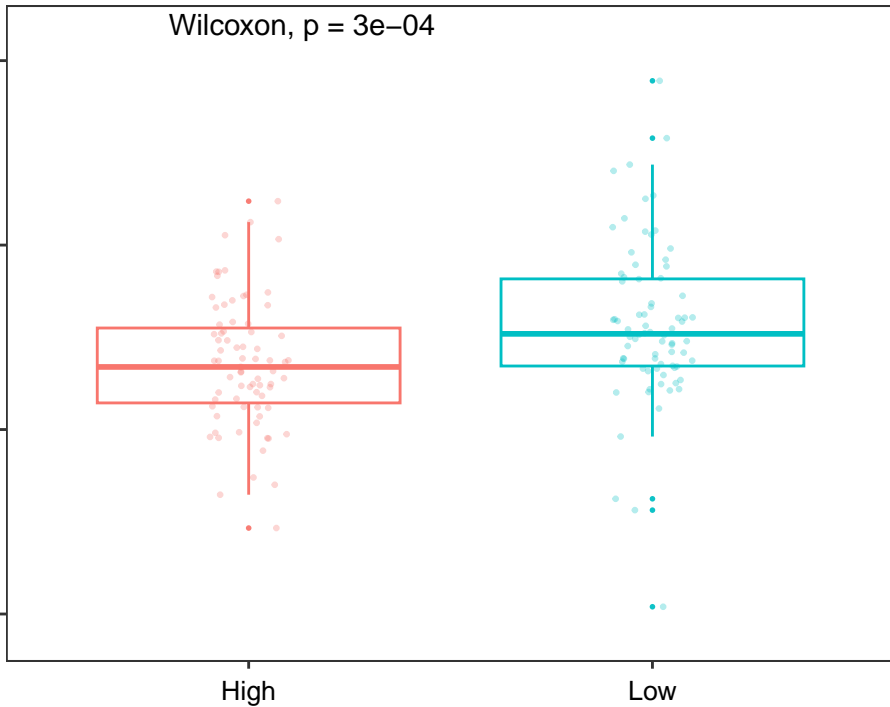

Supplement: Supplementary file 5 [file DataSheet_2.zip › expression/MTDH.pdf]

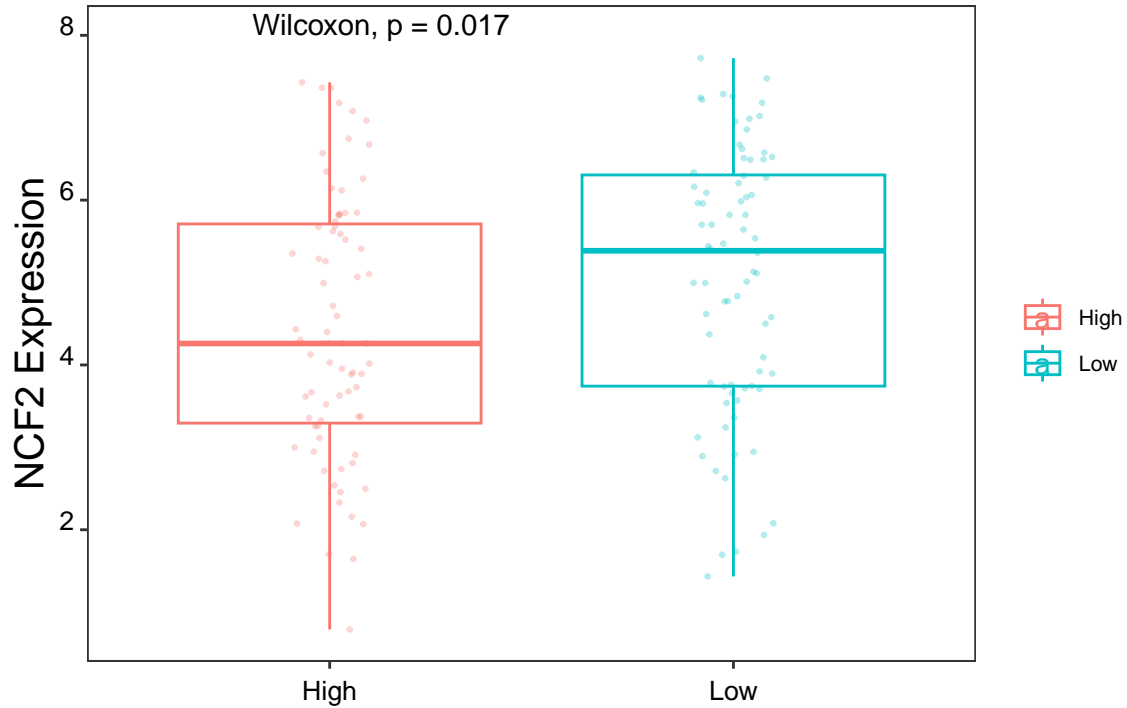

Supplement: Supplementary file 5 [file DataSheet_2.zip › expression/NCF2.pdf]

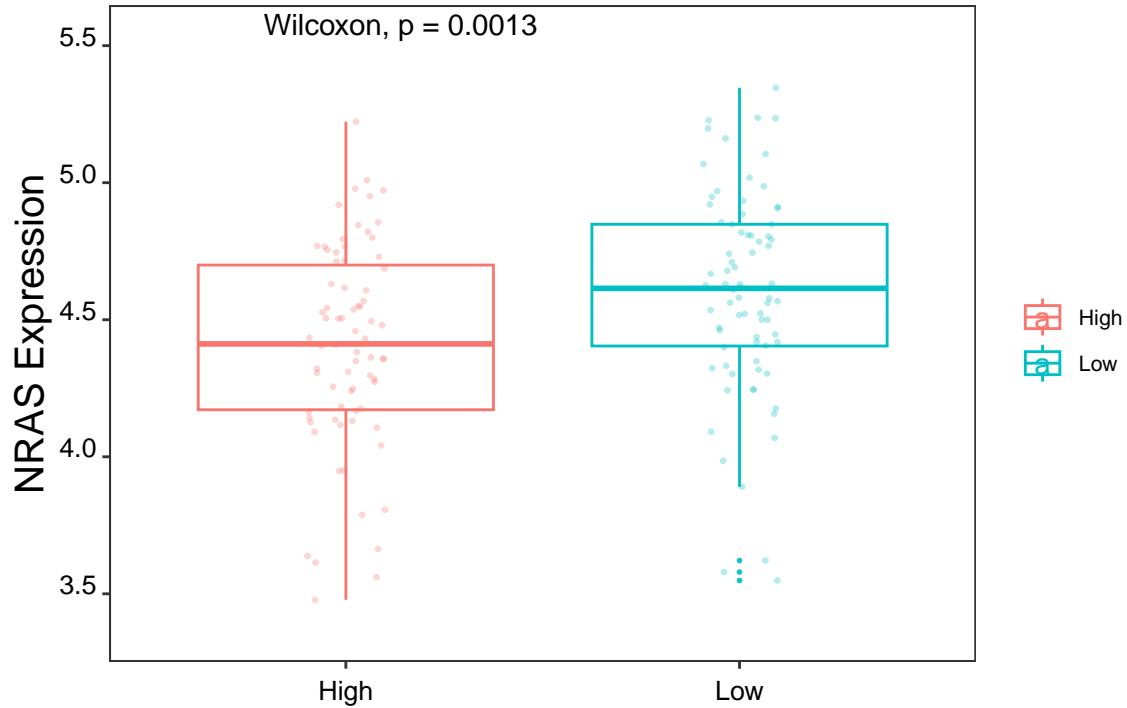

Supplement: Supplementary file 5 [file DataSheet_2.zip › expression/NRAS.pdf]

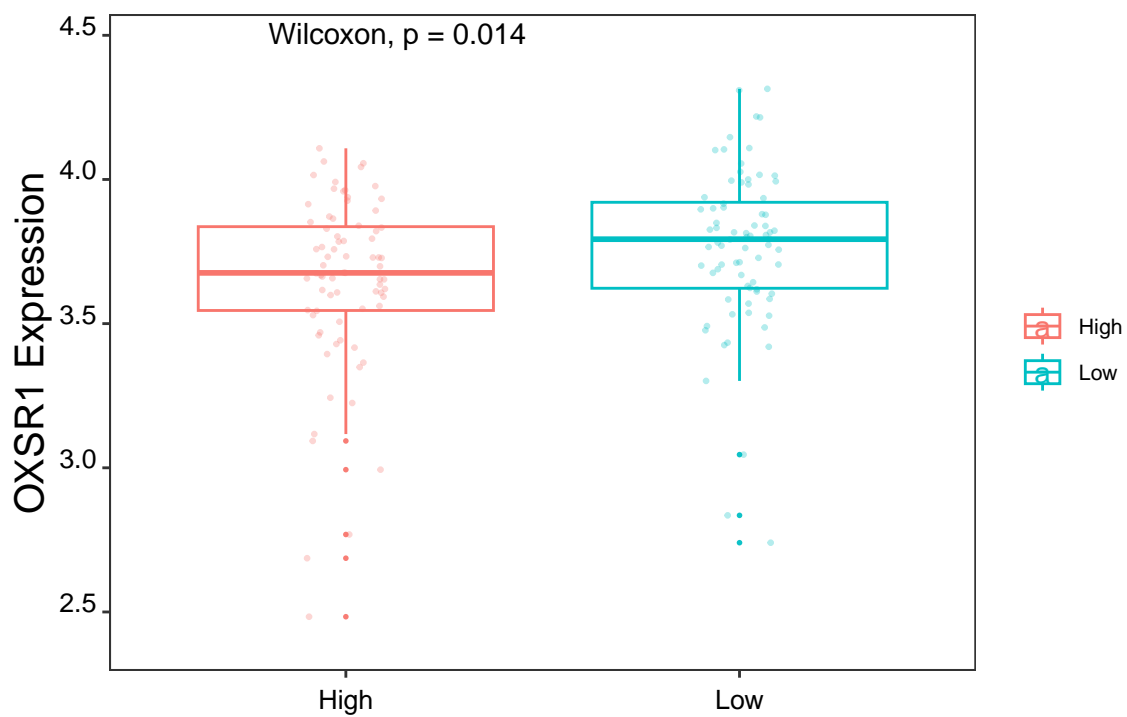

Supplement: Supplementary file 5 [file DataSheet_2.zip › expression/OXSR1.pdf]

Wilcoxon,  $p = 0.02$

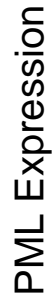

5

1

3

High

Low

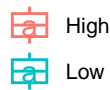

Supplement: Supplementary file 5 [file DataSheet_2.zip › expression/PML.pdf]

Wilcoxon,  $p = 0.056$

PRKAA1 Expression

5

4

3

High

Low

High  
Low

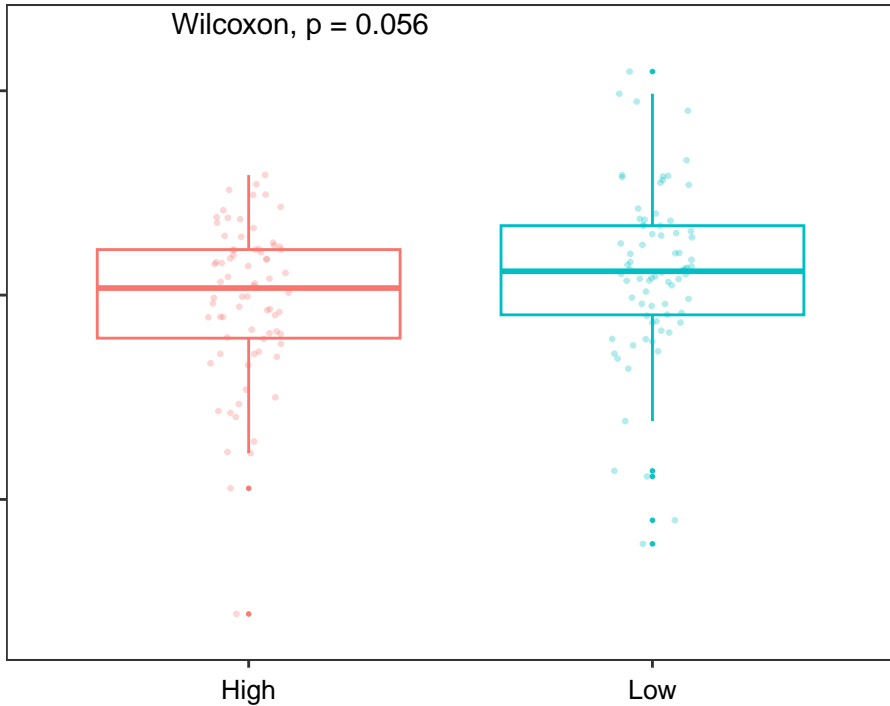

Supplement: Supplementary file 5 [file DataSheet_2.zip › expression/PRKAA1.pdf]

Wilcoxon,  $p = 0.0067$

RB1 Expression

6

5

4

3

2

High

Low

High  
Low

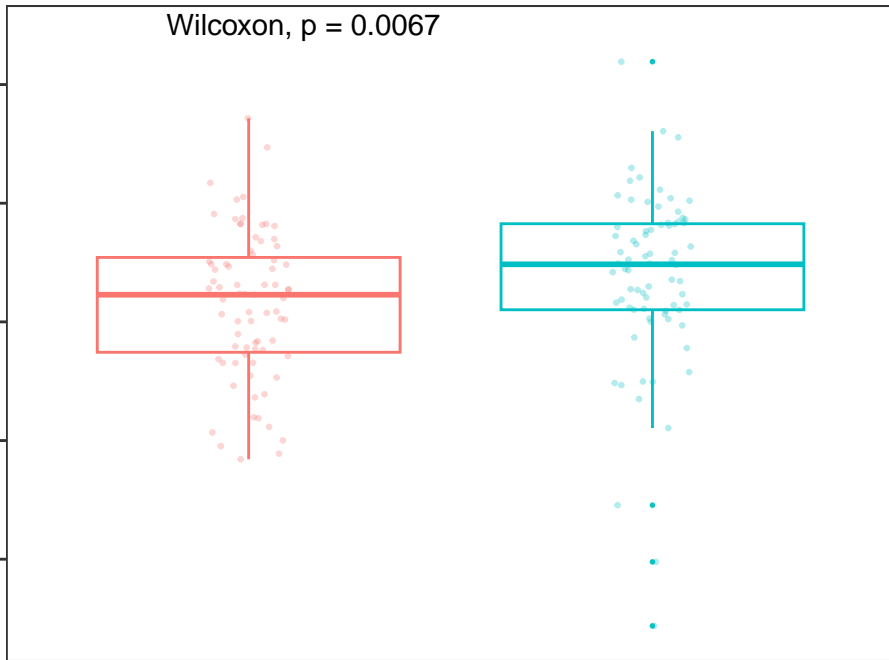

Supplement: Supplementary file 5 [file DataSheet_2.zip › expression/RB1.pdf]

Wilcoxon,  $p = 0.005$

SCP2 Expression

2

3

4

High

Low

High  
Low

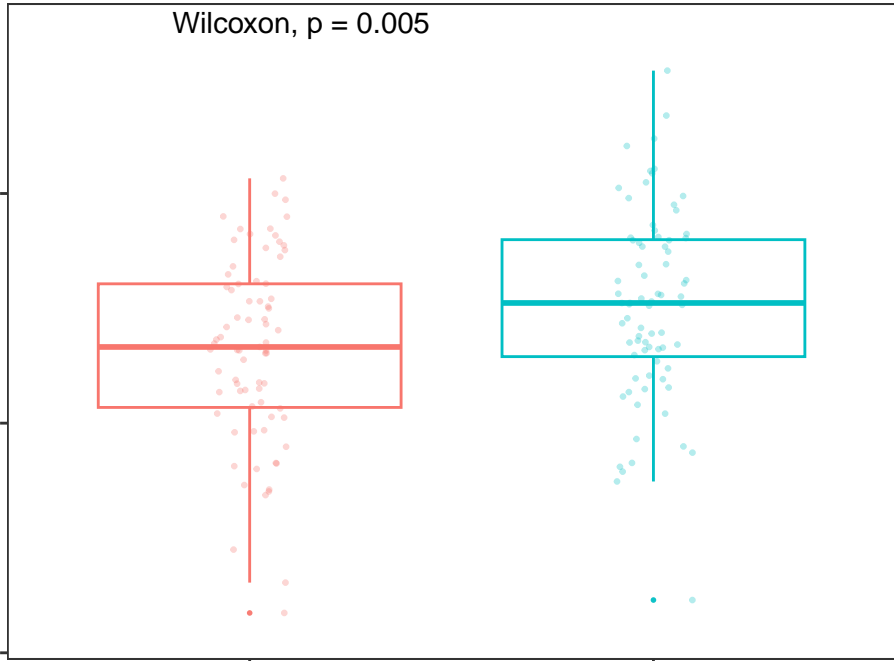

Supplement: Supplementary file 5 [file DataSheet_2.zip › expression/SCP2.pdf]

SETD1B Expression

Wilcoxon,  $p = 0.0045$

5

4

3

High

Low

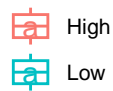

Supplement: Supplementary file 5 [file DataSheet_2.zip › expression/SETD1B.pdf]

Wilcoxon,  $p = 0.021$

SIRT1 Expression

4

3

High

Low

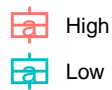

Supplement: Supplementary file 5 [file DataSheet_2.zip › expression/SIRT1.pdf]

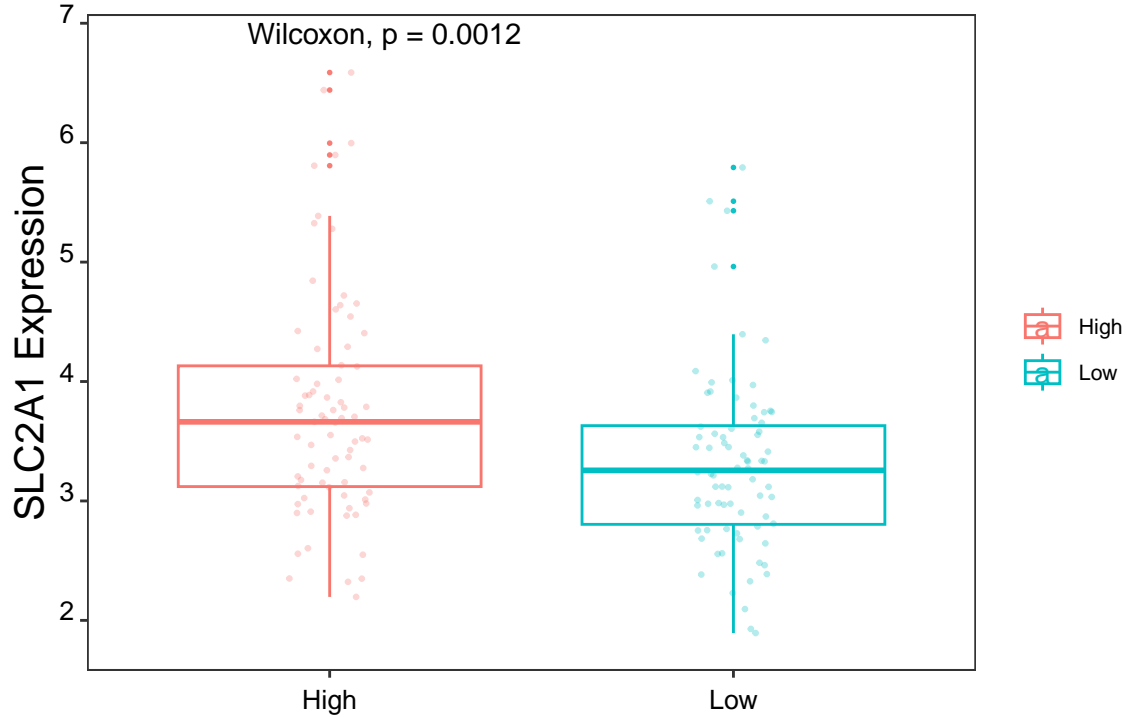

Supplement: Supplementary file 5 [file DataSheet_2.zip › expression/SLC2A1.pdf]

SLC3A2 Expression

Wilcoxon,  $p = 0.054$

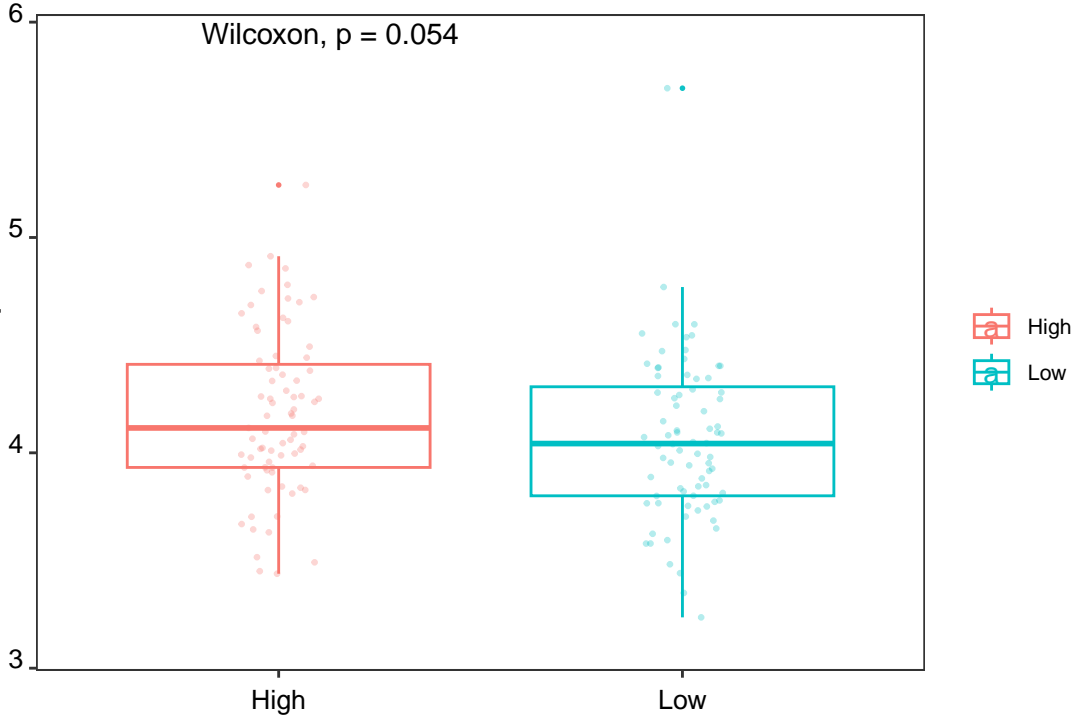

Supplement: Supplementary file 5 [file DataSheet_2.zip › expression/SLC3A2.pdf]

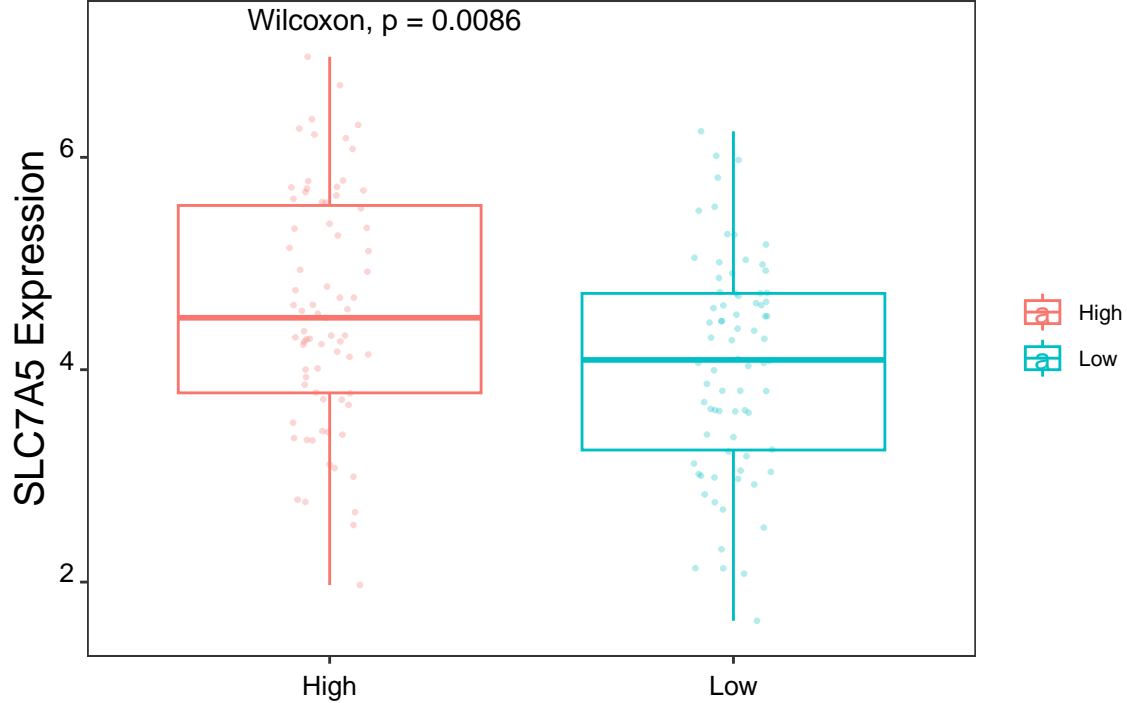

Supplement: Supplementary file 5 [file DataSheet_2.zip › expression/SLC7A5.pdf]

SNX4 Expression

Wilcoxon,  $p = 0.00017$

High

Low

High  
Low

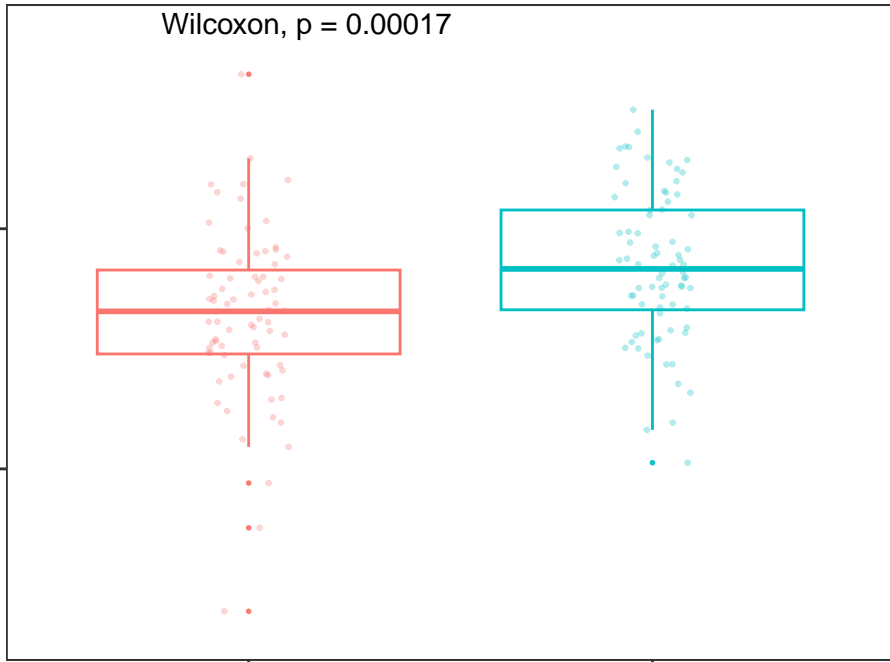

Supplement: Supplementary file 5 [file DataSheet_2.zip › expression/SNX4.pdf]

Wilcoxon,  $p = 0.021$

TLR4 Expression

6  
4  
2  
0

High

Low

High  
Low

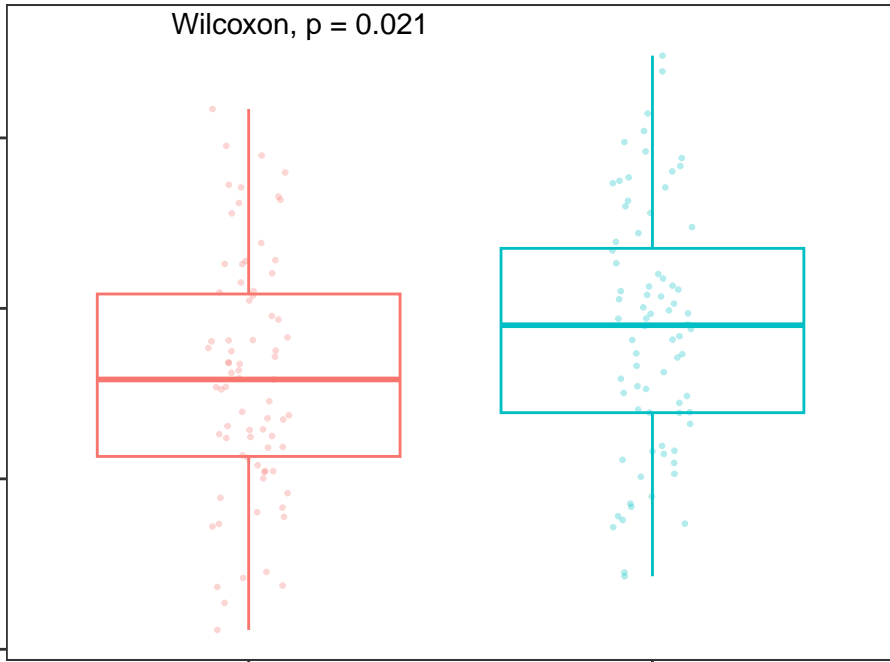

Supplement: Supplementary file 5 [file DataSheet_2.zip › expression/TLR4.pdf]

TMBIM4 Expression

Wilcoxon,  $p = 2e-04$

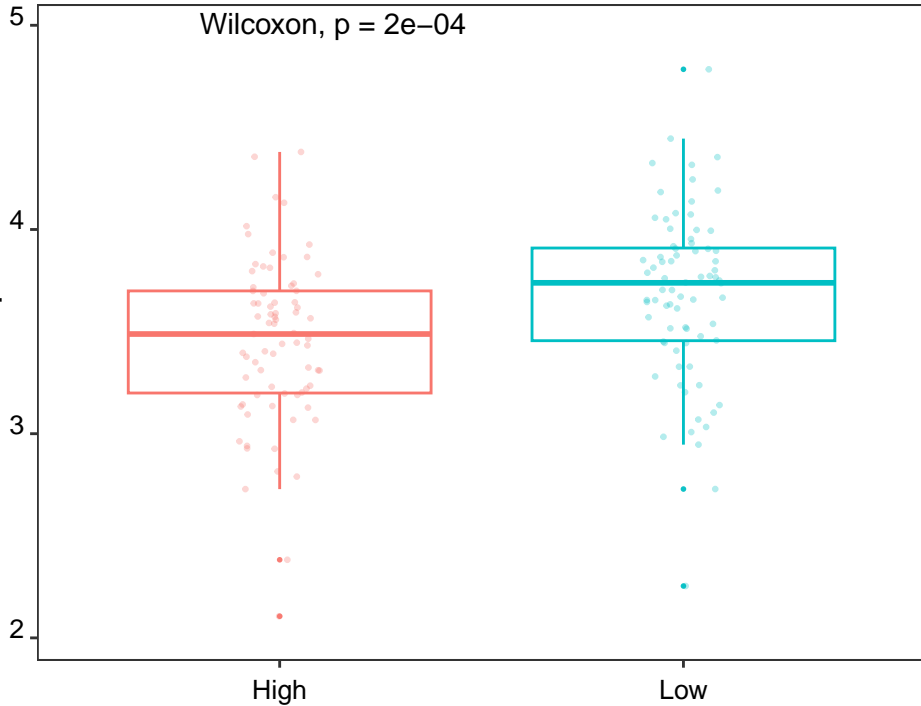

High  
Low

Supplement: Supplementary file 5 [file DataSheet_2.zip › expression/TMBIM4.pdf]

Wilcoxon,  $p = 0.0061$

TXNRD1 Expression

5.0  
4.5  
4.0  
3.5  
3.0

High

Low

High  
Low

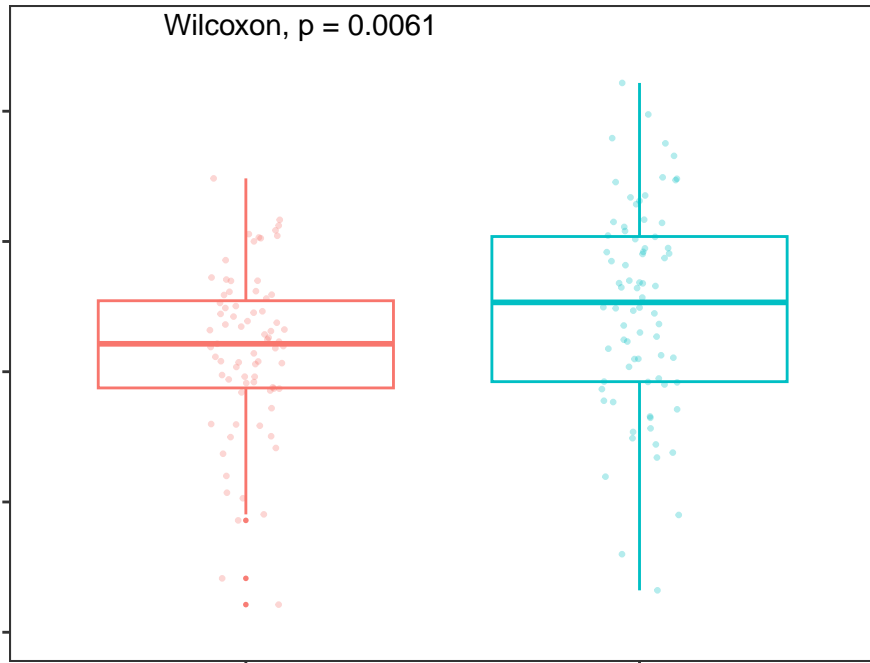

Supplement: Supplementary file 5 [file DataSheet_2.zip › expression/TXNRD1.pdf]

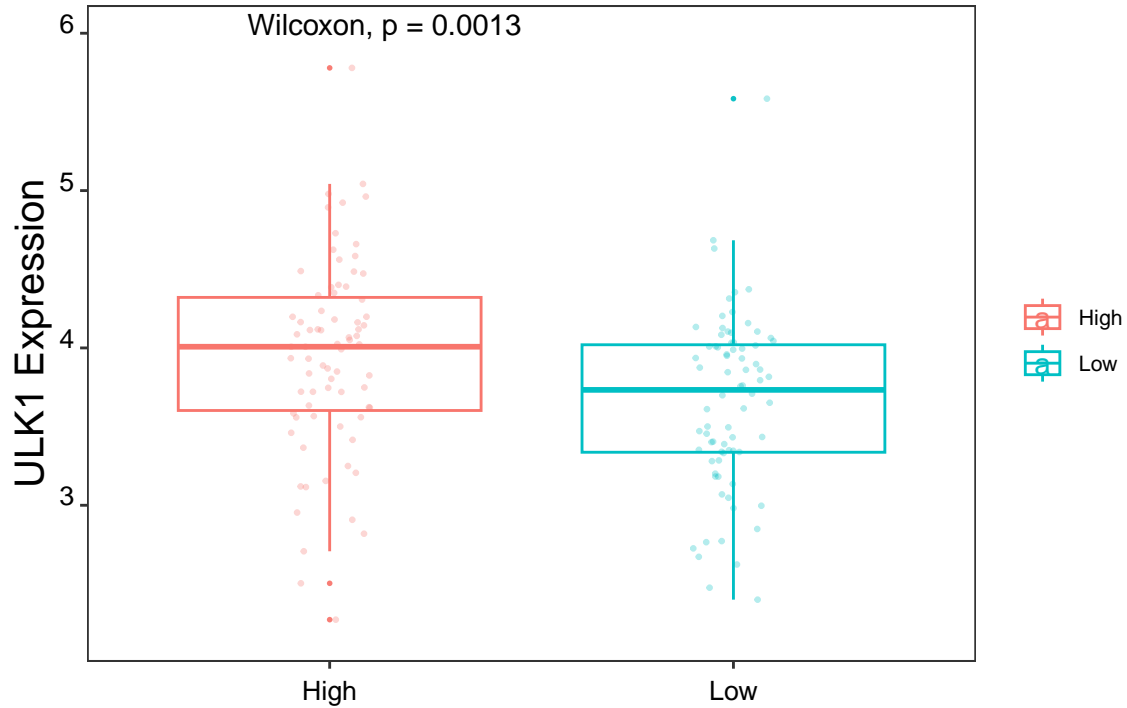

Supplement: Supplementary file 5 [file DataSheet_2.zip › expression/ULK1.pdf]

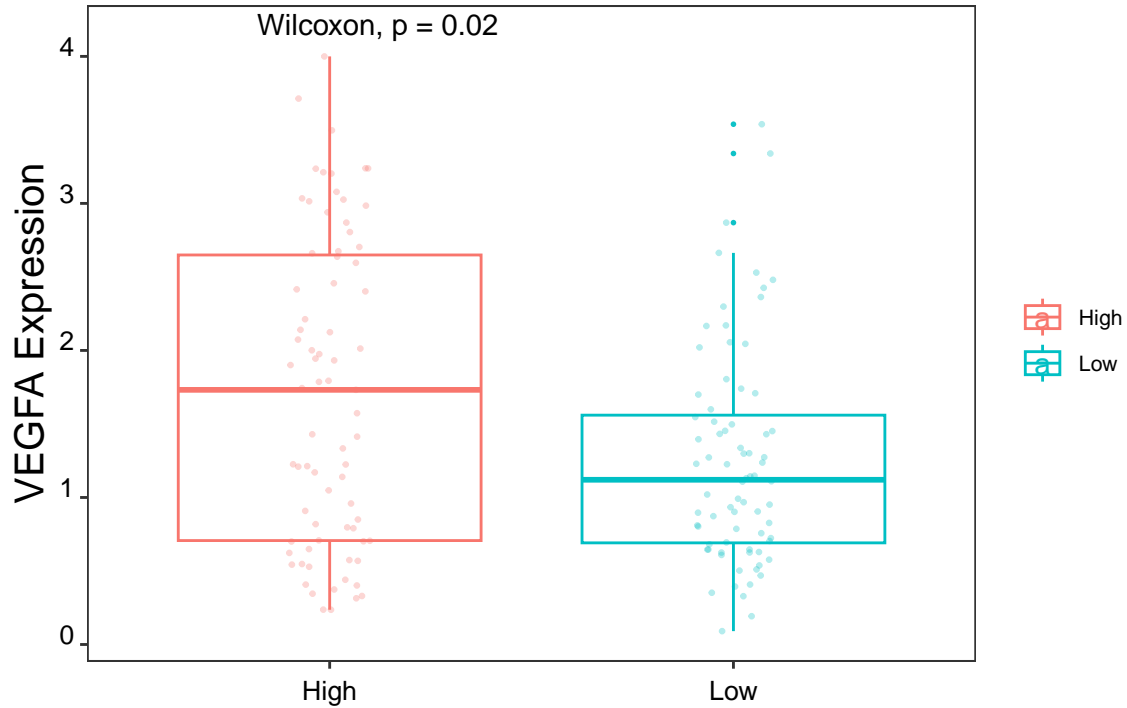

Supplement: Supplementary file 5 [file DataSheet_2.zip › expression/VEGFA.pdf]
