# Supplementary material for: Identification and validation of a novel CD8+ T cell-associated prognostic model based on ferroptosis in acute myeloid leukemia
Source: Front Immunol. 2023 Apr 17;14:1149513. doi: 10.3389/fimmu.2023.1149513 (PMC10150955; doi:10.3389/fimmu.2023.1149513)
Supplement: Supplementary file 4 [file DataSheet_1.docx]

**SUPPLEMENT**

**Supplementary Notes**

For **Supplementary Figure 1S**, the collection of AML samples has been introduced in MATERIALS AND METHODS. The AML samples were thawed and stained with APC/Cyanine7 anti-human CD8 (Biolegend) and FITC anti-human CD3 (Biolegend) to enumerate CD8 T cell subset (CD3+CD8+). Staining was performed according to the published protocol(1). Stained cells were detected on BD LSRFortessa™ X-20 Cell Analyzer (BD Biosciences).

**Supplementary Figure Legends**

**Supplementary Figure 1S. Deconvolution and enumeration of CD8+ T cell subset in 6 AML samples. (A)** Scatterplots of flow cytometry enumerating CD8+ T cells in AML samples (CIBERSORT deconvolution data were labeled on the bottom of each scatterplot). **(B)** Direct comparison between CIBERSORT and flow cytometry. Concordance was determined by Pearson correlation (R) and linear regression (solid lines). *p* <0.05 was defined as significant.

**Supplementary Figure 2S.** Heatmap showing correlations between CD8+ T cell-related ferroptosis genes from Gene6 risk score and 22 major immune cell types in AML. **p* < 0.05, ***p* < 0.01, ****p* < 0.001.

**Supplementary Figure 3S.** Correlation between signature gene expression and immunotherapeutic response in AMLs. *p* < 0.05 was defined as statistically significant.

**Supplementary References**

1. Maecker HT, Rinfret A, D'Souza P, Darden J, Roig E, Landry C, et al. Standardization of Cytokine Flow Cytometry Assays. *BMC Immunology* (2005) 6(1):13. doi: 10.1186/1471-2172-6-13.
